# Organ-on-a-Chip Applications in Microfluidic Platforms

**DOI:** 10.3390/mi16020201

**Published:** 2025-02-10

**Authors:** Ling An, Yi Liu, Yaling Liu

**Affiliations:** 1 School of Engineering, Dali University, Dali 671003, China; anling@stu.dali.edu.cn; 2 Precision Medicine Translational Research Center, West China Hospital, Sichuan University, Chengdu 610041, China; 3 Department of Bioengineering, Lehigh University, Bethlehem, PA 18015, USA

**Keywords:** microfluidics, organ-on-a-chip (OoC), drug development, disease modeling, personalized medicine

## Abstract

Microfluidic technology plays a crucial role in organ-on-a-chip (OoC) systems by replicating human physiological processes and disease states, significantly advancing biomedical research and drug discovery. This article reviews the design and fabrication processes of microfluidic devices. It also explores how these technologies are integrated into OoC platforms to simulate human physiological environments, highlighting key principles, technological advances, and diverse applications. Through case studies involving the simulation of multiple organs such as the heart, liver, and lungs, the article evaluates the impact of OoC systems’ integrated microfluidic technology on drug screening, toxicity assessment, and personalized medicine. In addition, this article considers technical challenges, ethical issues, and future directions, and looks ahead to further optimizing the functionality and biomimetic precision of OoCs through innovation, emphasizing its critical role in promoting personalized medicine and precision treatment strategies.

## 1. Introduction

**Figure 1:**
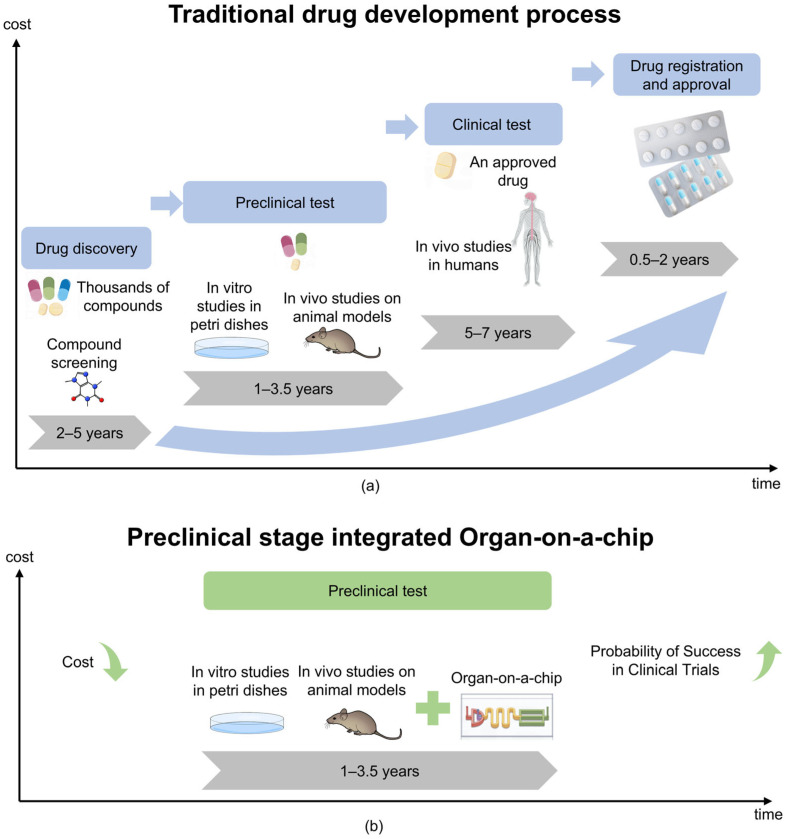
Comparison of traditional models of drug development processes with integrated OoC technology. (**a**) Under the traditional model, the cost of drug development gradually increases over time. (**b**) The integrating of OoC technology is expected to reduce drug development costs and potentially increase the probability of clinical trial success.

Cells are the fundamental unit of life, and their microenvironment plays a crucial role in regulating proliferation, migration, and differentiation [1,2,3]. Traditional in vitro models, including 2D cell cultures and animal models, struggle to replicate the complexity of human tissues [4,5]. Two-dimensional cultures lack dynamic physiological conditions. Animal models, on the other hand, present ethical concerns, incur high costs, and often fail in clinical translation—nearly 60% of drugs tested in animals do not succeed in human trials [6,7]. Furthermore, as shown in Figure 1a, the development of new drugs is a challenging and costly endeavor. These limitations drive the need for advanced bioengineered systems to better mimic organ functions.

Three-dimensional cell culture technology improves physiological relevance but lacks optical accessibility and fluid dynamics, making cellular analysis challenging [8,9]. Organ-on-a-chip (OoC) technology, based on microfluidics, has emerged as a promising alternative [10,11]. These systems precisely recreate cellular microenvironments. They incorporate fluid flow, mechanical forces, and biochemical gradients at physiologically relevant scales [12,13]. Though technically complex, microfluidic OoC models significantly enhance disease modeling, drug screening, and personalized medicine [14]. Table 1 summarizes the advantages and disadvantages of each model.

Organs-on-a-chip (OoC), also called microphysiological systems (MPS) [15,16,17], are small-scale biomimetic cell culture devices. They integrate three-dimensional tissue engineering with microfluidic technology [18,19]. This system simulates key factors in organ physiology, including perfusion and mechanical stress. It achieves this through a continuously perfused micron-scale microfluidic network, effectively replicating the structure and function of human organs [20]. OoC designs usually feature multiple controllable parallel channels. They also include precisely configured pumps, valves, and integrated sensors for electrical and biochemical monitoring. These components allow human cells to be cultured in dynamic environments with precise control over biological processes [21]. OoCs provide a high-fidelity human cell model through these technologies, combined with precise control of environmental conditions. As technology has advanced, the fidelity of these systems has increased significantly, enabling the performance of thousands of quantitative assays at high-throughput, single-cell resolution [22]. This advanced microfluidic OoC helps simulate key three-dimensional functional units of human organs, such as tissue–tissue interfaces between specialized endothelial and parenchymal cells and organ-specific mechanical movements (e.g., respiratory movements of the lungs or intestines) [16,17]. In addition, it allows replication of physiological functions at the cellular or organ level, providing insights into pharmacokinetics (PK) and pharmacodynamics (PD) in humans without relying on animal models [18]. The OoC industry is growing rapidly, and many pharmaceutical and biotech companies have begun to use this technology to quickly obtain patient-specific data, bringing unprecedented opportunities for personalized medicine and drug development [19]. In addition, as shown in Figure 1b, the application of OoCs can accelerate the drug development process and help reduce related Research and Development costs.

In addition to replicating organ-level physiology, recent advancements in artificial intelligence (AI), machine learning (ML), and 3D bioprinting have driven the next generation of OoC platforms [23,24]. AI-driven optimization of microfluidic chip designs has enhanced fluid dynamics modeling, improving drug transport predictions. ML algorithms now aid in real-time analysis of cellular responses within OoC devices, enabling automated toxicity assessments and precision drug screening [25,26]. Moreover, 3D bioprinting has revolutionized OoC fabrication, allowing the construction of vascularized tissue structures with unprecedented spatial precision. These interdisciplinary approaches are transforming OoCs from a static in vitro model into a dynamic, adaptive system with predictive power. This transformation is opening new frontiers in biomedical research.

While significant progress has been made in the development of microfluidic-based OoC technologies, several critical gaps remain that limit their broader application. The lack of standardized fabrication protocols hinders reproducibility and cross-laboratory validation. Additionally, the limited long-term viability of cultured tissues challenges their use in chronic disease modeling. The integration of real-time data analytics, particularly leveraging advanced machine learning techniques, is still in its early stages. Ethical and regulatory frameworks for clinical translation are also underdeveloped. Addressing these challenges in the next 5–10 years through standardized manufacturing, better biomimetic materials, and AI integration will be essential for advancing OoC platforms to routine clinical use and personalized medicine.

This review provides a comprehensive study of microfluidics-based OoC technology, focusing on material selection, fabrication strategies, advantages, and emerging applications. Through detailed case studies, it explores how these devices simulate specific organs, such as the heart, liver, and lungs. It also highlights their applications in cancer therapy. Additionally, this review highlights emerging trends, such as ML-enhanced chip optimization. It also discusses multiplexed OoC systems that mimic multi-organ interactions. It further delineates the significant influence of microfluidic integration on pivotal areas of biomedical research, such as drug screening, disease modeling, toxicity evaluation, and personalized medicine. In addition, this review explores existing research gaps and discusses future development trends as well as challenges and opportunities.

## 2. Fundamentals of Microfluidics

This section includes two parts. The first part discusses the advantages of microfluidic technology in OoCs and its application advantages in emerging fields. The second part discusses several commonly used microfluidic chip manufacturing methods, and analyzes the impact of material selection on the performance and application of OoCs.

### 2.1. Microfluidics in OoCs: From Core Advantages to Cutting-Edge Innovations

Microfluidic technology offers precise control of fluid flow, enabling efficient manipulation of fluids at the microscale. It is ideal for high-throughput screening due to its small size and reduced reagent use [8,9]. By integrating mechanical forces like shear and strain, microfluidic chips can simulate physiological conditions, such as blood flow in vascular models, and support long-term cell culture by accurately supplying nutrients and oxygen [10,11]. These systems are used in diverse applications, including cell sorting, biochemical analysis, and OoC models. Furthermore, microfluidic devices can replicate multi-organ systems, facilitating the study of drug absorption, distribution, metabolism, and excretion (ADME), as well as disease mechanisms through barrier models that simulate physiological functions without compromising cell viability [12,13].

In addition to the advantages of microfluidics technology mentioned above that have been fully confirmed in previous reviews (such as precise fluid control and high-throughput screening), recent breakthroughs are driving its expansion into more cutting-edge fields and showing obvious advantages in emerging fields. The microfluidics-based OoC platform not only realizes single-cell omics analysis [27], highly biomimetic organoid culture [28], and multi-organ interaction modeling [1], but also gives rise to intelligent and personalized organ-on-a-chip systems through deep integration with artificial intelligence algorithms (optimizing fluid behavior prediction) and 3D bioprinting (constructing vascularized heart chips) [23,24,29,30]. In the field of immunology research, microfluidics technology provides a high-throughput screening platform for immunotherapy by simulating the tumor immune microenvironment (such as T cell infiltration and PD-1 inhibitor response) [31]. In addition, its clinical translation potential is particularly prominent in personalized medicine: patient-derived organoids (such as glioblastoma chips) can be used to customize chemotherapy regimens [30], while environmental toxicology applications (such as microfluidic lung chips to evaluate the toxicity of microplastics) have opened up a new paradigm for environmental health research [32].

### 2.2. Microfluidic Chip Manufacturing Methods and Material Selection for OoCs

There are several microfluidic chip fabrication methods suitable for making OoCs. Each method has its own unique advantages and applicable material selections. Material selection is critical in the development of microfluidic devices and can significantly affect their functionality and reliability. A wide variety of materials can be used to create microfluidic systems. These include traditional silicon and glass, various polymers such as flexible rubbers, thermosets, and thermoplastics, and even paper for cost-sensitive and disposable applications. The choice of materials depends on the specific needs of the application. It also considers the required functionality and the level of integration with other systems [33,34]. Factors such as flexibility, breathability, electrical conductivity, solvent resistance, optical clarity, and biocompatibility are critical during the design phase. Additionally, cost-effectiveness is crucial, especially for mass production of disposable microfluidic devices to minimize cross-contamination risks. Therefore, material selection is closely related to several factors. These include the performance of the microfluidic system, the economic feasibility of the manufacturing process, and the overall success of the application [35]. The following introduces several common microfluidic chip manufacturing methods and material selection.

**Figure 2:**
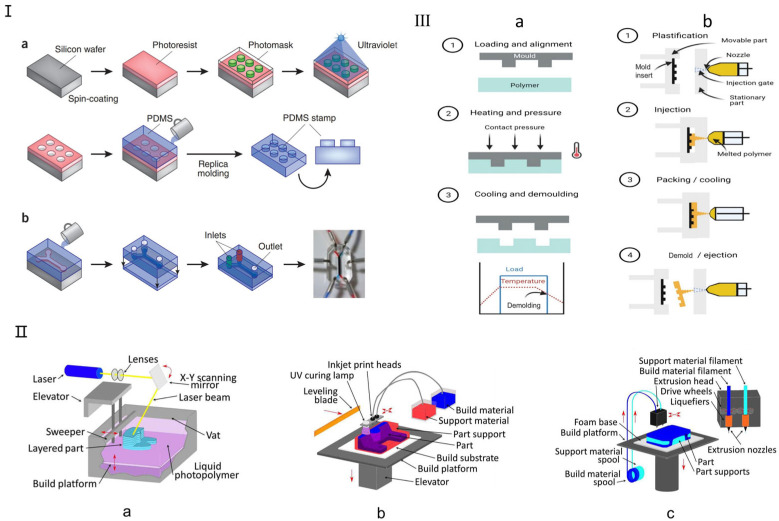
Traditional fabrication methods for microfluidic chips. (**I**) Soft lithography microfluidic chip manufacturing method. (**a**) Soft lithography creates PDMS stamps by coating a silicon chip with a photoresist, overlaying it with a patterned photomask, and exposing it to UV light. The process involves washing away exposed areas to form microscale patterns, which are then used for microcontact printing on substrates, including microfluidic devices. (**b**) A single-channel microfluidic device is made by sealing a PDMS stamp with two inlets and one outlet onto a glass substrate. The device, shown with red and blue dyes, uses side channels to periodically alter the membrane for cellular studies. Reproduced with permission from [19]. (**II**) Three-dimensional Printing Microfluidic Chip Fabrication Method. (**a**) Stereolithography (SL); (**b**) Multi Jet Modeling (MJM); (**c**) Fused Deposition Modeling (FDM, also termed “thermoplastic extrusion”). Reproduced with permission from [29]. (**III**) Schematic for (**a**) hot embossing and (**b**) micro-injection molding. Reproduced with permission from [36].

Currently, the primary traditional manufacturing methods for microfluidic chips include soft lithography, hot embossing, and injection molding, each suited to different fabrication needs (Figure 2). Soft lithography, introduced by Whitesides in 1998, enables the replication of microstructures on silicon chips using biocompatible materials like polydimethylsiloxane (PDMS) and polymethyl methacrylate (PMMA) [33,34]. PDMS offers excellent optical clarity, allowing real-time, high-resolution imaging of cellular responses (Figure 2I), and supports the fabrication of complex 3D microenvironments via replica molding. Other established methods include hot embossing (Figure 2III(a)), which shapes thermoplastics like PC, PMMA, and COP under heat and pressure, offering high precision and cost efficiency, making it ideal for commercial-scale organ chip production [36,37]. Injection molding (Figure 2III(b)), developed in the 1980s, enables high-throughput production of uniform microfluidic chips by injecting molten thermoplastics into molds. Despite its high initial costs, it remains a key technique in commercial OoC fabrication due to its efficiency and reproducibility [36,38]. Recent advancements have further enhanced its fluid-handling capabilities, improving microfluidic functionalities.

Three-dimensional printing is a promising alternative to traditional microfluidic fabrication, offering rapid production, cost-efficiency, and the ability to create complex structures [39]. Unlike PDMS-based devices that require manual labor and bulky control systems, 3D printing allows layer-by-layer fabrication from a digital model, enabling flexible design modifications and rapid prototyping [40]. It also eliminates the need for a cleanroom environment, with relatively low-cost consumables like resins and solvents. However, not all 3D printing technologies are suitable for microfluidics [41]. The most relevant techniques include stereolithography (SL) (Figure 2II(a)), multi-jet modeling (MJM) (Figure 2II(b)), and fused deposition modeling (FDM) (Figure 2II(c)), which enable precise microfluidic channel fabrication using photosensitive resins and biocompatible polymers tailored for customized organ chips [29]. Recent advancements have expanded 3D printing applications in biomedical microfluidics. Gowers et al. developed the first wearable 3D-printed microfluidic device, integrating an FDA-approved microdialysis probe and a needle-shaped biosensor for continuous metabolite monitoring, with potential for real-time athlete training evaluation [42]. Bishop et al. [43] used stereolithography to create a 3D-printed microfluidic biosensor capable of measuring electrochemiluminescence signals. With its ability to precisely manufacture complex structures, 3D printing is increasingly used in microfluidic organ chips, particularly SL and FDM technologies for optimized channel fabrication.

However, multiple emerging fabrication approaches are reshaping the field by addressing limitations in complexity, scalability, and biomimicry. Laser-cut synthetic biomaterials, recent advances in biofabrication, leverage laser-patterned synthetic hydrogels to guide organoid self-organization. Gjorevski et al. [44] demonstrated geometry-driven intestinal organoid patterning using PEG-based matrices, while Lutolf’s group pioneered light-guided stereolithography for creating spatially controlled stem cell niches [45]. These materials enable dynamic stiffness gradients (1–50 kPa) matching native tissues while permitting optical interrogation. CNC-milled reconfigurable systems, mechanical machining techniques, exemplified by Beebe’s group’s modular platforms, utilize polymethylmethacrylate (PMMA) or polycarbonate (PC) to create open-top microfluidics [46]. CNC milling achieves channel resolutions down to 50 μm, supporting real-time imaging of paracrine signaling and cell migration. Paper-based 3D microenvironments, cellulose matrices, offer low-cost (<$0.10/chip), hypoxia-gradable platforms for tumor modeling. Rodenhizer et al. [47] (Nat. Mater. 2016, 15.2) developed paper-based tumor chips that recapitulate metabolic heterogeneity through controlled oxygen diffusion (0.1–21% gradient). Through Roll-to-Roll Manufacturing [48], the manufacturing efficiency of microfluidic chips has been significantly improved by mass-producing microstructures on continuous flexible substrates. Flexography [49], the transfer of patterns to substrates using flexible printing plates, can be used to make epoxy molds. This method provides a way to quickly manufacture microfluidic devices. Microthermoforming [50] is a method of molding heated thermoplastic polymer films into microstructures. This technology can be used to make three-dimensional microfluidic structures with freely suspended microcavities, such as chips for cell culture. Focused Ion Beam Milling [51] involves the use of focused ion beams to perform precise micro-nanoscale processing on the surface of materials and can be used to manufacture high-precision microfluidic channels and structures. Laser Micromachining [52] uses high-precision lasers to etch microstructures on a variety of materials, and is suitable for rapid prototyping and the manufacture of complex microfluidic structures. These emerging technologies provide a variety of options for the manufacture of microfluidic chips, showing unique advantages in terms of precision, speed, and material compatibility.

Table 2 summarizes the contents of all the microfluidic chip manufacturing methods mentioned above, including traditional manufacturing methods and emerging manufacturing methods, including fabrication complexity, time required, specialty equipment needed, costs, application advantages, and limitations.

## 3. Case Studies of Microfluidic Applications in OoCs

The human body constitutes a dynamic system, encompassing numerous organs and intricate physiological microenvironments. Integrating OoCs with cutting-edge technologies to facilitate interactions between organs and their microenvironments remains a key focus in scientific research [53,54,55]. To address this challenge, researchers have drawn insights from various disciplines, such as physics, chemistry, biology, and engineering, to develop OoC technology. Fields like materials science, microelectronics, and medicine have also contributed significantly. At the heart of this technology lies the utilization of microfluidic technology to construct a microphysiological system on a chip [56,57]. Using OoCs within a microfluidic environment, instead of traditional two-dimensional cell cultures, allows researchers to create comprehensive in vitro systems. These systems can emulate multiple organs, biofluids, mechanical signals, and functional tissue interfaces [58]. The objective of OoCs is to replicate the architecture and functionality of human organs. This includes processes like pulmonary respiration and cardiac pulsation [59,60]. This groundbreaking technology holds transformative potential in many fields, such as cell biology, personalized medicine, and drug development. It also plays a crucial role in cancer diagnosis and therapy. This review provides an overview of several OoC systems that have been designed, focusing on research in drug screening and disease modeling, and explores related topics such as pharmacology and cancer research [61,62]. Furthermore, it delves into the challenges, opportunities, and prospective developments of these systems. At the same time, we also explore different cases of combining organ chips with ML to enhance the innovation of the article. Table 3 summarizes different types of OoC technologies, including their structural characteristics, key materials, application areas, challenges and limitations, and cutting-edge applications combined with machine learning (ML) and deep learning (DL), demonstrating the broad potential of OoCs in biomedical research, drug screening, disease modeling, and personalized medicine. Specific details are given in the following introduction.

### 3.1. Lung-on-a-Chip

The lung, as the principal respiratory organ of the human body, possesses a structure that is both complex and intricate. This organ consists of a network of airways, including the trachea, bronchi, and bronchioles, alongside numerous alveoli [63]. Serving as the fundamental unit of gas exchange, the alveoli facilitate the exchange of oxygen and carbon dioxide through a specialized interface within the respiratory membrane [64]. Consequently, the in vitro biomimetic reconstruction of the lung and its microenvironment presents a formidable challenge, necessitating interdisciplinary collaboration across fields such as anatomy, physics, materials science, cell biology, and tissue engineering [65]. With technological progress, particularly advancements in micromachining and miniaturization, OoC technology grounded in microfluidics has garnered significant attention. This variant of OoCs represents a miniaturized cell culture device capable of precisely emulating the functions of organs and tissues in vitro [66,67]. As a trailblazer in the OoC domain, the lung-on-a-chip introduces innovative approaches for mimicking the lung cell microenvironment and crafting lung disease models [68,69,70]. It is anticipated to advance the evaluation of pulmonary medications, toxicity testing, and the formulation of disease models, thus fostering fundamental scientific research and its translation into clinical practice.

**Figure 3:**
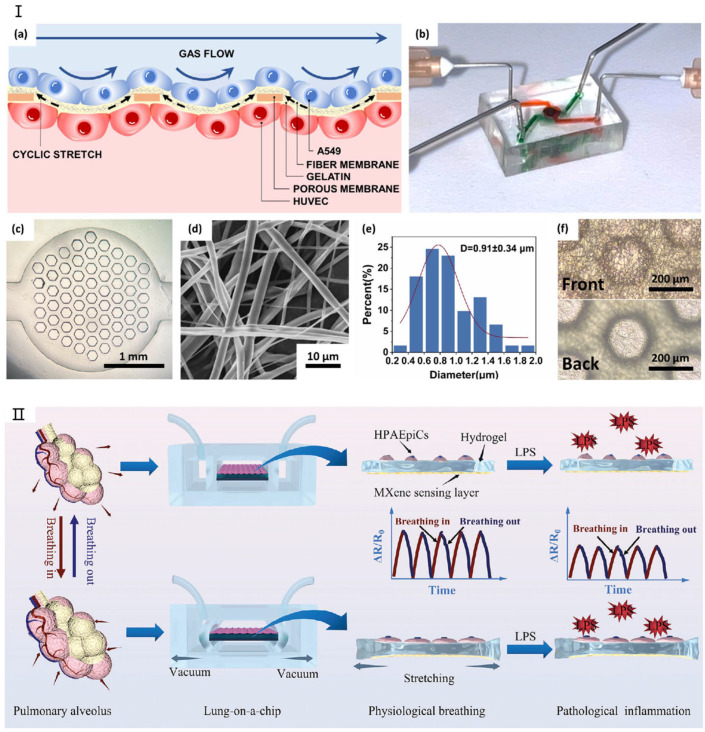
The latest research on lung-on-a-chip. (**I**) (**a**) Schematic illustration of the SBS nanofiber/porous PDMS composite membrane structure. (**b**) Photograph of the lung-on-a-chip device. (**c**) Optical microscopy image showing the porous PDMS composite membrane bonded to the lower channel. (**d**) Scanning electron microscopy (SEM) image depicting an SBS nanofiber. (**e**) Diameter distribution analysis of SBS nanofibers. (**f**) Comparison of the front and back surfaces of the composite membrane. Reproduced with permission from [78]. (**II**) Real-time monitoring system for lung-on-a-chip. Reproduced with permission from [79].

Lung models utilizing microfluidic chip technology typically feature a layered microchip structure [71,72,73]. The inaugural lung chip, developed by Huh et al. [74] was fabricated from polydimethylsiloxane (PDMS) and effectively replicated the human respiratory process. Recent advancements include lung chips fabricated from alternative materials, like the lung/airway chip developed by Humayun et al. [64] from poly(methyl methacrylate) to replicate the lung bronchus’s microenvironment. Moreover, Tavana et al. [69] explored fluid accumulation-induced damage to lung epithelial cells by designing a microfluidic lung chip, while Douville et al. [62] replicated the mechanical stretching characteristics of the human alveolar air–blood barrier with a novel microfluidic device. Amid the urgent need for effective treatments for coronavirus disease 2019 (COVID-19), caused by severe acute respiratory syndrome coronavirus 2 (SARS-CoV-2), which impacts multiple tissues including the lungs, the lung chip based on deep learning developed by Sun et al. [66] has been applied in COVID-19 infection research. Figure 3 shows in detail the latest progress of lung-on-a-chip. In addition, Zamprogn et al. [75] proposed a lung chip that simulated a series of tiny alveoli with in vivo-like sizes based on biological, stretchable, and biodegradable membranes made of collagen and elastin. They achieved reconstruction of the air–blood barrier using primary alveolar epithelial cells and primary pulmonary endothelial cells. Shen et al. [76] replaced the PDMS membrane with a thin, biocompatible, soft, and stretchable F127-DA hydrogel-based membrane that closely resembles the composition and stiffness of the extracellular matrix of human alveolar cells for the construction of a lung chip. The chip well reconstructed the mechanical microenvironment within the alveoli, so that epithelial/endothelial functions were highly expressed in the alveolar–capillary barrier. The chip can reflect the synergistic effects of matrix stiffness and tensile strain on the occurrence of pulmonary fibrosis, providing a new approach to studying lung diseases such as COVID-19 infection and fibrosis [77].

### 3.2. Liver-on-a-Chip

**Figure 4:**
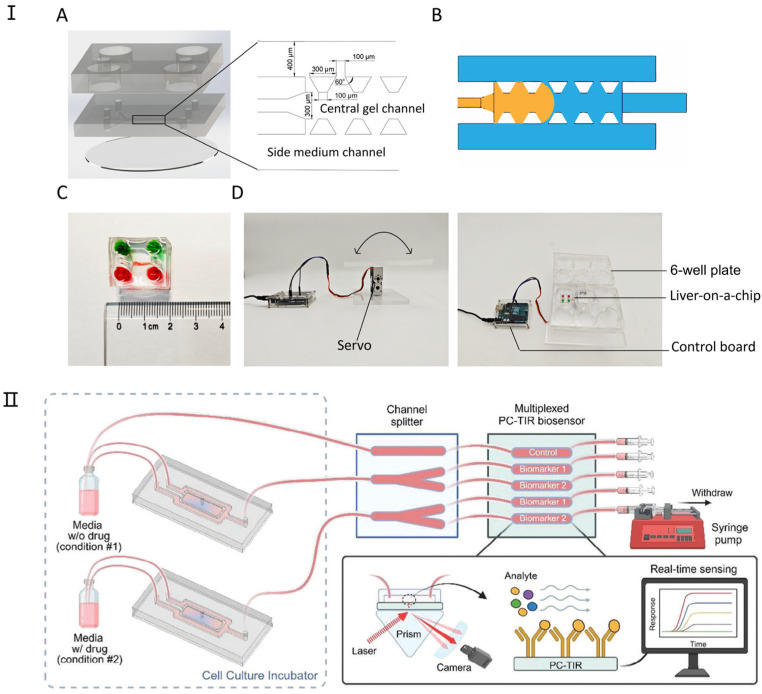
The latest research on liver-on-a-chip. (**I**) Design and components of the liver-on-a-chip. (**A**) The liver-on-a-chip consists of three main layers: a medium reservoir layer, a three-channel cell culture layer, and a glass base. (**B**) A schematic illustration of fluid dynamics within the chip, where the liquid (orange) remains confined to the central channel upon injection due to surface tension. (**C**) An assembled liver-on-a-chip filled with dye for visualization. (**D**) A custom-built programmable rocker used for gravity-driven perfusion in the liver-on-a-chip system. Reproduced with permission from [84]. (**II**) Integration of a 3D liver-on-a-chip with a PC-TIR Optical Biosensor for Real-Time Monitoring of Liver Biomarkers. This microfluidic system combines a 3D liver-on-a-chip model with a microchannel splitter chip and a PC-TIR biosensor, enabling the detection of protein biomarkers in solution. This setup facilitates rapid, continuous, and multiplexed monitoring of liver-secreted biomarkers, providing a real-time assessment of drug-induced toxicity in organ-on-a-chip models. Reproduced with permission from [85].

The liver is an intricate organ responsible for executing a multitude of metabolic functions. It plays a pivotal role in regulating energy expenditure; synthesizing bile, hormones, and plasma proteins; and metabolizing exogenous substances [80,81]. Despite its notable regenerative capabilities, sustained disease states or viral challenges may culminate in irreversible functional degradation. Investigations into the pathology of liver diseases frequently concentrate on pharmaceutical development and the bioengineering of liver tissues, encompassing a range of in vitro experimental models [82]. However, conventional liver models encounter significant constraints, catalyzing the advancement of liver-on-a-chip and microbioreactor technologies capable of furnishing meticulously controlled microenvironmental conditions. Leveraging microtechnologies enables the utilization of minimal human cell quantities to replicate the liver’s complex internal milieu and microarchitecture across two- and three-dimensional settings, offering a promising avenue for liver tissue engineering and the evolution of organ systems [83]. These microtechnology-driven methodologies are instrumental in augmenting liver-specific functions and facilitating applications that employ various 3D culture strategies, essential for preserving liver functionality and emulating its natural characteristics. Figure 4 shows in detail the latest progress of liver-on-a-chip.

Among various organs, the liver is particularly amenable to microtechnology applications due to its intricate microstructure and the nuanced interactions within its environment. Liver-on-a-chip technology not only facilitates predictions of the toxicological impacts and metabolic pathways of pharmaceuticals on the liver but also enables integration with chips mimicking other organs. This integration forms a system that replicates the comprehensive human body response, allowing for an all-encompassing evaluation of drug effects [86]. The incorporation of microtechnology in liver research offers several benefits, including the following: (1) the enhancement of liver functionality within simulated authentic physiological contexts; (2) the fabrication of complex microscale structures that replicate the natural liver architecture, which is crucial for liver tissue engineering; and (3) the use of a minimal number of human cells to streamline and optimize the screening of various treatments and toxic substances [87]. For instance, researchers like Banaeiyan [81] have designed and fabricated a large-scale liver lobule chip device to create a microphysiological environment conducive to liver cell growth. Similarly, Lu et al. [87] developed a simulated 3D liver tumor chip employing microfluidic technology alongside a decellularized liver matrix (DLM) and gelatin methylacrylate (GelMA) within a dynamic three-dimensional cell culture system. This system more accurately emulates the tumor microenvironment, encompassing matrix proteins, growth factors, optimal stiffness, and fluid shear stress to support cell proliferation, thereby demonstrating its efficacy in replicating the liver’s complex milieu.

### 3.3. Heart-on-a-Chip

**Figure 5:**
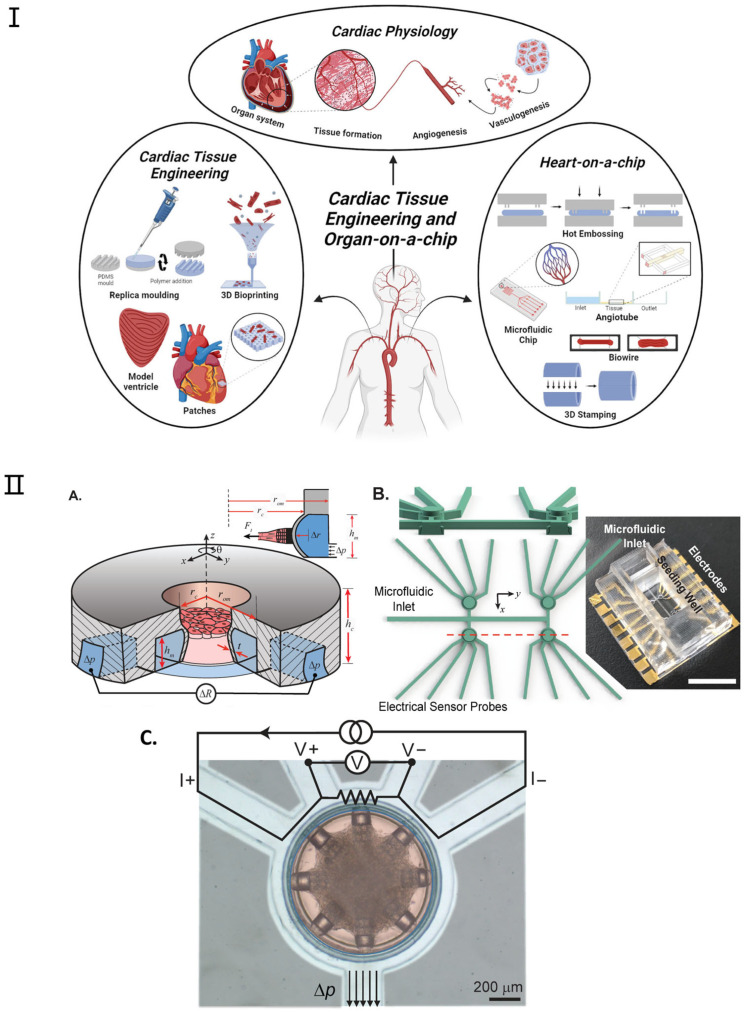
Examples of heart-on-a-chip platforms. (**I**). Overview of the applications and fabrication techniques within heart tissue engineering and heart-on-a-chip. Reproduced with permission from [94]. (**II**) (**A**) Platform Overview: The device features a cylindrical seeding well for cardiac microtissues and an annular microchannel for mechanical actuation and sensing. External pressure (Δp) bends the microchannel, allowing force detection through changes in electrical resistance (ΔR). (**B**) 3D Mold Design: The figure includes a cross-sectional view of the seeding wells and a photograph of the completed platform. Device cavities and microfluidic channels are isolated, with a large top opening for cell pipetting and microfluidic inlets connected to annular channels. Scale bar: 10 mm. (**C**) False-Colored Top View: The figure displays a device with cardiac microtissue in the seeding well (red) and connecting microchannels (light blue). The annular microchannel serves as an electrical sensor using a four-wire resistance circuit. Applied pressure (Δp) results in measurable strain. Scale bar: 200 μm. Adapted with permission from [89].

Recent advancements have been achieved in the development of microscale engineered heart tissue, colloquially known as heart-on-a-chip systems, dedicated to exploring heart physiology and pathology. These heart models are constructed by amalgamating human cells, micromachining technology, and state-of-the-art sensors to emulate the human heart’s architecture and functionality within a microfluidic setting [88,89]. Anticipated to catalyze a paradigm shift in drug testing, disease modeling, personalized medicine, and the management of cardiovascular diseases, these systems promise to unveil novel pathways for efficacious interventions. Heart-on-a-chip systems furnish a tissue-like milieu, enabling meticulous regulation of specific variables [90]. Contemporary research utilizing these platforms offers scientists the prospect to delve into human tissue biology and conduct high-throughput studies [91,92,93,94]. Figure 5 shows the application and fabrication technologies for cardiac tissue engineering and heart-on-a-chip. For instance, Thavandiran et al. [91] utilized a three-dimensional matrix-based microenvironment and PDMS pillars to fabricate cardiac microtissues; similarly, the I-wire system devised by Sidorov et al. [92] employed two parallel PDMS pillars to organize cardiac tissue neatly upon cell seeding. More recently, Jayne et al. [89] introduced a microfluidic platform designed for the precision construction of cardiac microtissues within a rigorously controlled microenvironment (as shown in Figure 5II). Lind et al. [93] introduced a simple method to fabricate novel instrumented heart physiological devices through multi-material 3D printing. Specifically, they designed six functional inks based on piezoresistive, high-conductance, and biocompatible soft materials that can integrate soft strain gauge sensors into microstructures to guide the self-assembly of biomimetic layered cardiac tissue.

Additionally, the integration of biosensors into organ-on-chip platforms could enable the next-generation heart-on-a-chip to continuously monitor dynamic tissue behavior in real time. Environmental parameters such as pH, temperature, and dissolved oxygen could also be monitored with integrated analytical devices [94,95]. Among various applications, drug-induced cardiotoxicity, especially chemotherapeutic drugs, is the most studied concept reviewed through biosensors integrated into cardiac tissue platforms. However, the detection of disease progression and enhanced myocardial mechanical function are some other demonstrative topics being investigated. This field can be subdivided into the monitoring of cardiomyocyte contractility and extracellular field potential [96,97]. So far, electrochemical and optical biosensors are the main transducers that have been incorporated into the platform; however, other transducers have also been used. Among other techniques, optical methods, including microscopy, are the most commonly used methods to monitor cell contractions in real time. However, electrochemical sensors are more promising in revealing cell metabolism. These sensors are also better candidates for building miniature/portable chips [98].

### 3.4. Gut-on-a-Chip

**Figure 6:**
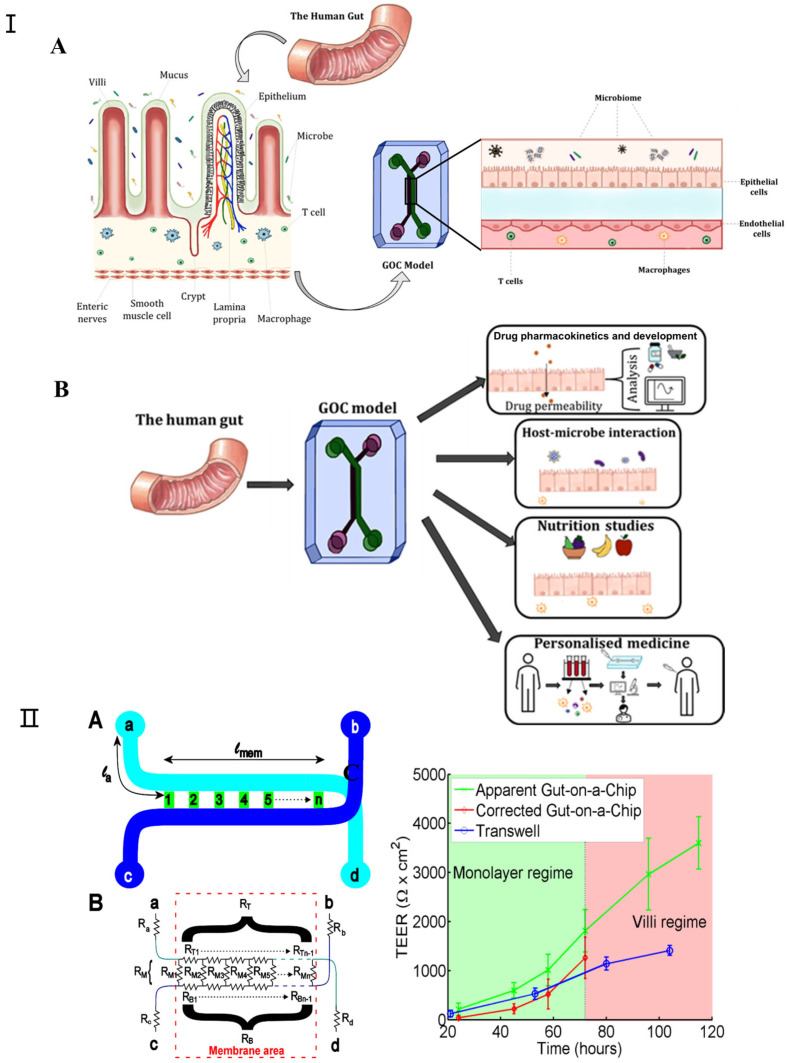
(**I**) (**A**) Schematic representation of GOC model mimicking the microenvironment of the gut. The human gut is covered with finger-like villi structures and invagination. (**B**) The various applications for which GOC models can be utilized, including drug pharmacokinetics and development, studying host–microbe interactions, nutrition and metabolism studies, and personalized medicine. Adapted with permission from [104]. (**II**) Transepithelial electrical resistance (TEER) measurement in gut-on-a-chip platform: (**A**) chip layout, (**B**) equivalent electrical circuit for the chip, (**C**) comparison of TEER measurements values for the gut-on-a-chip (green line) and transwell (blue line) human intestinal epithelial Caco-2 cells. The corrected gut-on-a-chip line is indicated as red line. Reproduced with permission from [105].

In recent years, gut-on-a-chip technology has witnessed significant advancements as an in vitro microfluidic platform that replicates human intestinal physiology, pathology, and pharmacology [99,100]. The evolution of this technology has garnered widespread acclaim, attributable not solely to the intestine’s fundamental role in human health but also to the critical interactions between the intestine, its resident microbiota, and other bodily organs [101]. Gut-on-a-chip devices are adept at emulating the human intestine’s structure and functionality within microfluidic environments, positioning them as viable substitutes for animal models in drug testing, disease modeling, and personalized therapy research. These systems incorporate biosensors for the real-time monitoring of pertinent physiological metrics, including intestinal barrier permeability, dissolved oxygen levels, cytokine profiles, and microbial production of short-chain fatty acids [102,103]. Such models extend beyond examining the digestive and absorptive capacities of the intestine, delving into its contributions to immunity, endocrine functions, and the resultant effects on other organ systems. While the gut-on-a-chip model introduces a novel approach to investigating gut and host–microbe dynamics, it faces certain constraints, such as its limited physiological relevance and the inadequate representation of endocrine and immune functionalities. Figure 6I [104] depicts a comprehensive system that integrates the gut-on-a-chip with its monitoring and culture apparatuses.

Since all the different mechanical factors can affect the barrier function of the intestinal epithelial layer, it is important to consider how this major function of the intestine can be assessed in a gut-on-a-chip. Furthermore, good barrier integrity improves confidence in the results of gut-on-a-chip studies; for example, in order to predict the oral bioavailability of drugs and the efficacy of treatments targeting leaky gut diseases such as IBD, measuring the barrier integrity of gut-on-a-chip devices is essential. For gut-on-a-chip designs, there are two approaches to measuring their barrier function: (1) assessing the transepithelial electrical resistance (TEER) or (2) using (labeled) tracer molecules to assess paracellular and/or transcellular permeability [106]. TEER is a non-invasive method used to characterize the barrier function of the cell layer formed within a gut-on-a-chip [107,108,109]. Srinivasan et al. extensively investigated the variation in TEER values due to factors such as temperature, medium formulation, cell passage number, and measurement techniques. Their review also analyzed the optimal parameters for TEER measurements and discussed the advantages and disadvantages of various measurement methods [110]. Odijk et al. again emphasized that additional factors must be considered when comparing the TEER values of different platforms by comparing measurements from organ chips and transwell models (Figure 6II) [105]. In their comparison, they noted that TEER measured in the gut-on-a-chip model was consistently higher than in static controls (Figure 6). Lucchetti et al. demonstrated the design, fabrication, and integration of thin-film electrodes into the HuMiX (human microbial crosstalk) platform to measure transepithelial electrical resistance (TEER) as a direct readout of real-time barrier tightness [109]. Vera et al. proposed a gut-on-a-chip model based on permeable hydrogel channels to perform TEER measurements in real time to quantify the integrity and tightness of the intestinal epithelial barrier formed in a 3D gut-on-a-chip model [110].

### 3.5. Brain-on-a-Chip

**Figure 7:**
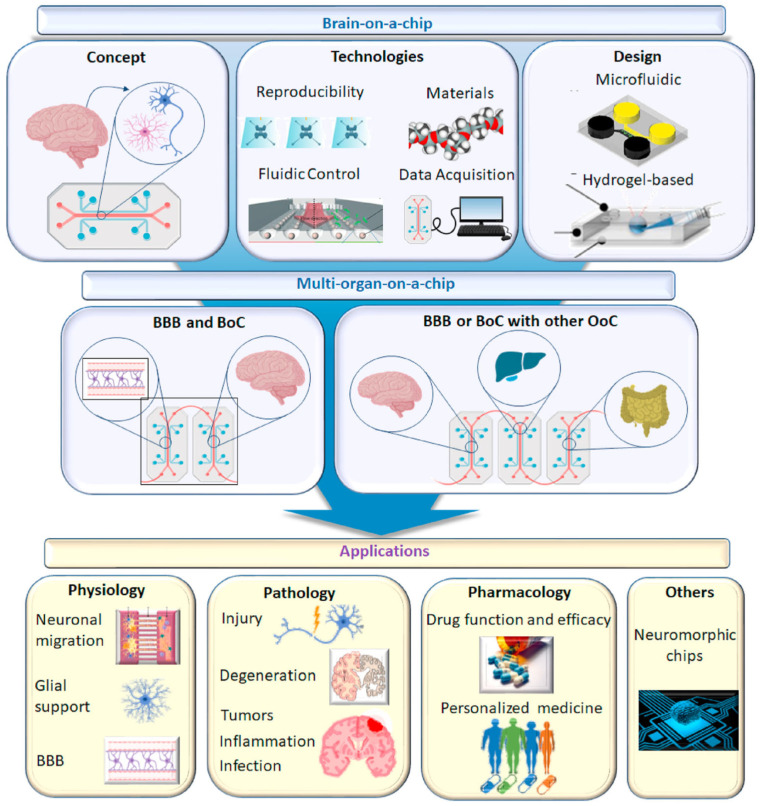
Schematic diagram of brain chip model and its application. Reproduced with permission from [112].

Brain-on-a-chip technology offers an innovative solution to the formidable challenges presented by the complexity of the human brain in the development of neurological medications. This advanced in vitro platform permits researchers to transcend the constraints of conventional methodologies by replicating essential components and functional attributes of the brain [111]. Initially, the term “brain-on-a-chip” referred to microfluidics-based systems featuring microengineered tissues; however, its definition has broadened to include a variety of in vitro central nervous system (CNS) modeling techniques [112]. The objective of brain-on-a-chip technology is to refine systems that adhere to specific benchmarks aiming to showcase a platform’s capacity to navigate the distinct challenges of in vitro CNS modeling, such as the simulation of the brain’s microenvironment and the incorporation of critical subunits like the blood–brain barrier, thereby adding significant value [113]. Employing human cells and dynamic systems, brain-on-a-chip models strive to generate microphysiological representations that mimic the functionality and architecture of specific brain regions. This includes compartmentalized neuronal compartments, either static or dynamic fluid conditions, the potential integration of non-neuronal cells (e.g., glial cells, vasculature), and biomaterial scaffolds [114]. With their micron-scale cell layer configurations, these models facilitate the formation of intricate neural networks within 3D cultures, capably capturing essential elements such as the blood–brain barrier (BBB), neurovascular units, neurons with their axonal synapses, and glial cells, thereby supporting their respective functions [115,116]. The employment of microfluidic technology in the fabrication of brain-on-a-chips marks a pivotal advancement in simulating the functions and structures of critical organs, highlighting the immense potential of these platforms in neuroscience research, disease modeling, and pharmaceutical development. Figure 7 shows a schematic diagram of the brain-on-chip model and its applications.

### 3.6. Kidney-on-a-Chip

**Figure 8:**
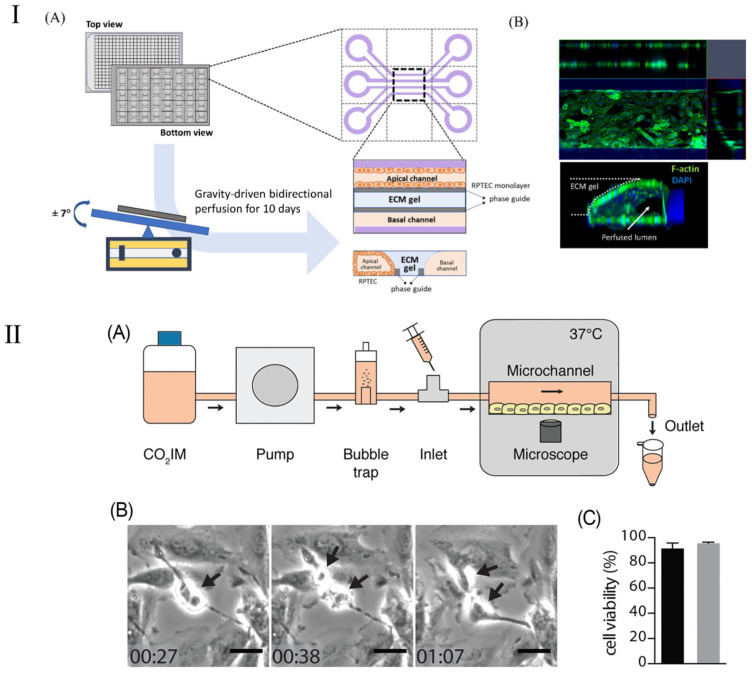
Schematic diagram of kidney chip model. (**I**) Schematic of a 3D tubular structure in a three-lane OrganoPlate. (**A**) The OrganoPlate, based on a 384-well plate, contains 40 microfluidic units. After 10 days of gravity-driven perfusion, a 3D tubular structure forms, with the basal channel filled with culture media. (**B**) Image showing 3D reconstruction of a kidney-on-a-chip unit. Reproduced with permission from [119]. (**II**) (**A**) Schematic representation of the PToC setup. (**B**) Representative frames from a time-lapse video showing an A498 renal epithelial cell dividing in the PToC (black arrows). Scale bar =15 μm, time = hh: mm. (**C**) Viability of A498 renal epithelial cells incubated in microchannels with CO2IM, under a flow rate of 75 μL/min (black) vs. static conditions (gray) for 8 h at 37 °C. Reproduced with permission from [120].

The kidney-on-a-chip represents a groundbreaking technological advancement engineered to replicate the functionality and internal milieu of the kidney, thereby surmounting the constraints inherent in traditional animal models utilized in pharmacological testing and disease investigation. The kidney chip not only has the potential to enhance drug dosing precision for the treatment of renal diseases but also offers researchers profound insights into the dynamics of blood urea and other nitrogenous waste products [117]. Furthermore, the deployment of kidney chips streamlines the processes of drug testing and development, empowering scientists to more efficaciously ascertain drug efficacy, detect drug-induced nephrotoxicity, and elucidate drug–drug interactions. By emulating the operational processes and mechanisms of the kidney, the kidney-on-a-chip emerges as a highly effective platform, rendering it exemplary for the execution of pharmacological evaluations [118,119]. This methodology is adept at producing extensive datasets, particularly through the determination of parameter values requisite for assessing drug effectiveness based on cellular analyses conducted within the chip [120]. Employing deep learning algorithms to scrutinize these parameters enables the categorization or prognostication of cellular responses to pharmacological agents within the chip, thereby determining drug efficacy [121,122]. This offers an innovative and efficient investigational tool for the management of renal pathologies. Kim et al. [117] reported on a pharmacokinetic study aiming to diminish the nephrotoxic effects of gentamicin within a perfusion kidney chip platform. This research elucidated the architecture of various kidney chip cohorts and the expression patterns of junctional proteins. Kim et al. [119] introduced a kidney chip model to evaluate renal injury caused by concentrated contrast media (CM) in the renal tubules, with a particular emphasis on the effects of contrast media viscosity and shear stress on renal tubular epithelial cells (Figure 8I). Their results showed that isotonic contrast media exhibited higher cytotoxicity under high shear stress conditions. This study highlights the importance of kidney chip models in simulating clinical scenarios and provides an innovative method to more accurately evaluate and understand the effects of contrast media in the kidney and their potential risks.

Although kidney-on-a-chip has shown significant promise in improving drug dosing accuracy and treating kidney disease, its applications are not limited to these areas. The kidney-on-a-chip’s high degree of customizability and functional replicability make it equally applicable to a wider range of kidney disease models, such as the study of kidney infection. Kidney infection, a severe form of urinary tract infection, mainly affects the renal pelvis and renal parenchyma of the kidney. Such infections are usually caused by bacteria, specifically Escherichia coli, which start at the entrance to the urethra, work their way up to the bladder, and eventually invade the kidneys [117,118]. In their study, Antypas et al. demonstrated a proximal-tubule-on-chip (PToC) model (shown in Figure 8II), which successfully simulated uropathogenic Escherichia coli (UPEC) behavior in the renal environment [120]. Through this model, the research team was able to observe the colonization process of UPEC on renal epithelial cells in detail, especially under simulated physiological shear stress conditions. This study provides important insights into understanding the microbial mechanisms of kidney infection, particularly how processes adapt to and resist physiological shear forces in a dynamic fluid environment. This discovery will help develop new strategies for kidney infections, especially in the prevention and treatment of urinary tract infections, and provides new targets and methods for future anti-infection treatments. In addition, this renal tubule chip model also demonstrates the potential of using microfluidic technology to study the interaction between pathogens and host cells, providing an effective tool for simulating more complex physiological and pathological conditions [119].

### 3.7. Cancer-on-a-Chip

The utilization of OoC technology in cancer research has emerged as a pivotal force in advancing precision medicine and personalized therapeutic approaches. This technology offers a biologically pertinent experimental platform for foundational research, pharmacological evaluation, and the development of cancer treatments by intricately mirroring the microenvironment of human organs, the complex interplay between cells, and biochemical gradients [123]. Particularly in emulating the tumor microenvironment, OoC technology adeptly reproduces the co-culture context of tumor cells and vascular endothelium, models the transendothelial migration of tumor cells, and their invasion into distant organs, furnishing a foundation for elucidating tumor genesis, metastasis, and treatment response. Furthermore, OoCs exhibit significant potential in investigating tumor aggressiveness, identifying new therapeutic targets, and assessing pharmacological effectiveness and toxicity [124,125]. Through the incorporation of patient-specific biological specimens and the application of deep learning algorithms to interpret the extensive datasets produced, OoCs additionally enable the tailoring and personalization of drug discovery and evaluation processes. This technology not only expedites the comprehension of cancer biology’s underlying mechanisms but also supplies robust experimental backing for the innovation and assessment of novel medications and the customized formulation of treatment regimens [126]. With ongoing technological progression and the broadening of its application spectrum, OoCs are anticipated to assume an increasingly vital and transformative role in the future landscape of cancer research and therapy. They present a unique opportunity to delve into human oncology with heightened accuracy and efficiency, all while circumventing animal testing, thereby inaugurating a new era in cancer treatment and drug development.

**Figure 9:**
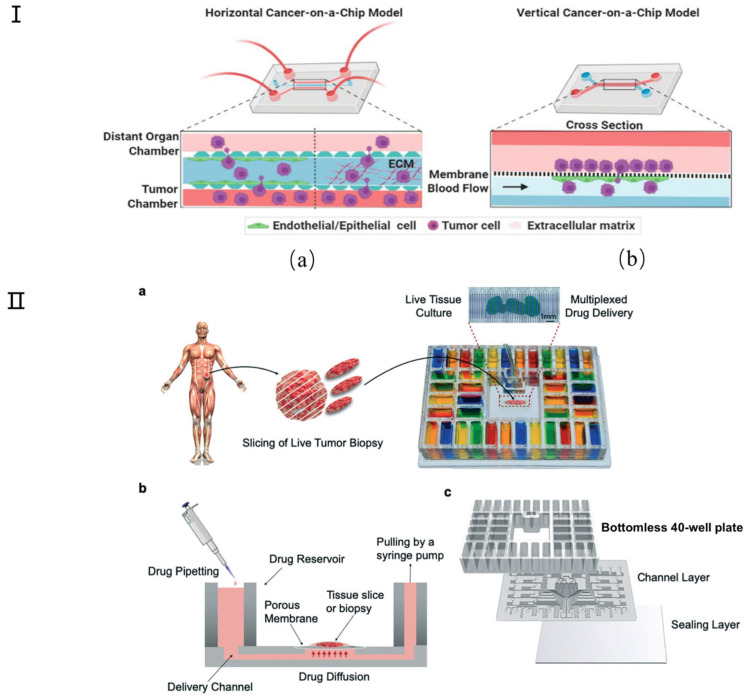
Schematic diagram of cancer chip model. (**I**) Simple representative microfluidic-based cancer-on-a-chip models show the movement of cancer cells from their site of origin to the distant organs. (**a**) In horizontal cancer chip models (such as the microvascular network (MVN) chip), the chambers are walled off by micron-sized pillars, which creates separate compartments for growing different cell types in their own zone without mixing with each other during the initial seeding, but which allow cellular interactions via paracrine, juxtracrine, or mechanical routes. (**b**) In vertical chips, the channels are separated by a membrane that may represent both cancer intravasation and extravasation processes. In some cases (such as ovarian TME organ-on-a chip (OTME)), a vertical layer can be integrated with the horizontal chips to mimic a more complex tumor pathophysiology. Reproduced with permission from [126]. (**II**) Microfluidic device design and overview. (**a**) Micrographs show a mouse glioma tumor slice treated with Hoechst (blue) and Sytox green (green) via alternating fluid streams. (**b**) The device uses gravity flow and a syringe pump to control flow across 40 fluidic streams. Tissue slices are cultured on a PTFE porous membrane that seals the open microchannels by capillarity, facilitating medium transport. (**c**) Exploded view of the PMMA platform includes a bottomless plate with 40 inlet wells, a 300 μm-thick channel network layer, and a 125 μm-thick sealing layer. Reproduced with permission from [128].

Many attempts have been made to develop physiologically relevant tumor chip models for cancer research. Simple representative microfluidic models, categorized into horizontal and vertical types, are employed to examine cancer models [127] (Figure 9I). Spheroids, as one of the most popular tumor chip models, remain the gold standard, meeting the need for simple in vitro tumor modeling. These models can mimic the organized cellular architecture present in solid tumors [128]. Microfluidic platforms have the potential to enable on-chip generation of primary tumor spheroids and therapeutic testing to match the unique tumor composition of each patient. For example, Nashimot et al. [123] have developed a tumor spheroid that integrates a perfusable vascular network to faithfully replicate in vivo tumor microenvironments (TMEs), facilitating nutrient, oxygen, and drug conveyance to the tumor via the vascular network. Although new microfluidic tumor chip platforms are constantly being developed for 3D tumor modeling, the utility of these devices depends on the technology to effectively analyze the resulting tumor models. Only in this way can biological information be collected for impactful translational research, including drug testing [124,125,126]. St-Georges-Robillard et al. used wide-field fluorescence hyperspectral imaging (HSI) technology to perform high-throughput analysis of ovarian tumor spheroids in microfluidic chips [127]. Tumor slices or microdissected tumor tissue can also be cultured on the tumor chip platform. For example, Rodriguez and colleagues developed a PMMA microfluidic device that can culture up to 40 patient-derived tumor tissue samples for drug screening (Figure 9II) [128]. Recently, researchers have also begun to investigate the use of tumor chip technology to evaluate combination therapies [129]. For example, Eduati et al. designed a droplet microfluidic platform to allow the generation of chemically different droplets. This has been used to perform paired drug testing on patient-derived pancreatic tumor cells, studying the effects of chemotherapy, targeted therapy, and cytokine combination therapy [130]. Other research groups have also explored the possibility of using tumor chip technology for personalized radiation therapy and immunotherapy. In immunotherapy, Aref and colleagues developed patient-derived organotypic tumor spheroid (PDOTS) chips to test the response to immune checkpoint blockade using antibody treatment [131,132].

### 3.8. Multiple Organs-on-Chip

There are two types of OoC: one is the single-organ system mentioned in our article above, which is used to simulate the key functions of a single tissue or organ; the second is a multiple organ-on-chip (MOC), which combines multiple organ chips together to reproduce the system interactions and responses of multiple organ models within a single system. MOCs connect single OoCs through pipes or microfluidic channels, usually wrapped in endothelial cells, or provide functional coupling by transferring effluent from the outflow reservoir of one organ to the input reservoir of another organ [133]. This MOC device includes various cell types cultured in independent chambers within a single chip, where each chamber represents a different organ. These chambers are connected by channels in a specific order according to the nature of the interaction between the organ and another organ in the body. This allows the effect of a drug on multiple organs to be studied simultaneously [134]. In either case, this coupling simulates the physiological coupling in vivo and provides appropriate cell–fluid volume ratios and flow distributions to create realistic in vitro models of human subsystems [135]. The most direct way to connect the functions of multiple organ systems is to collect the culture medium from the output of one platform and input it into another platform in sequence—for example, from the intestine to the liver to the kidney—to simulate the sequential adsorption, distribution, metabolism, and excretion of the compound, and then study the unexpected biological effects of metabolites in other organ systems [136,137,138]. The human chip system simulates the physiology of the whole organism by integrating many related single-organ models [139].

To be able to capture human physiology as well as possible, MOCs should feature [140] (1) human cells endogenously expressing main transporters and metabolizing enzymes; (2) organ models relevant for the exposure route; (3) individual organs-on-chip connected in a physiologically relevant manner; (4) a tight cellular barrier between the compartments; (5) organ models properly polarized in 3D; (6) the possibility for sampling in all major compartments; (7) construction from materials that do not absorb or adsorb the compound of interest; (8) cells that grow in the absence of fetal calf serum and Matrigel; (9) validation with a panel of compounds with known characteristics in humans; and (10) an integrated computer model translating concentrations to the human situation.

**Figure 10:**
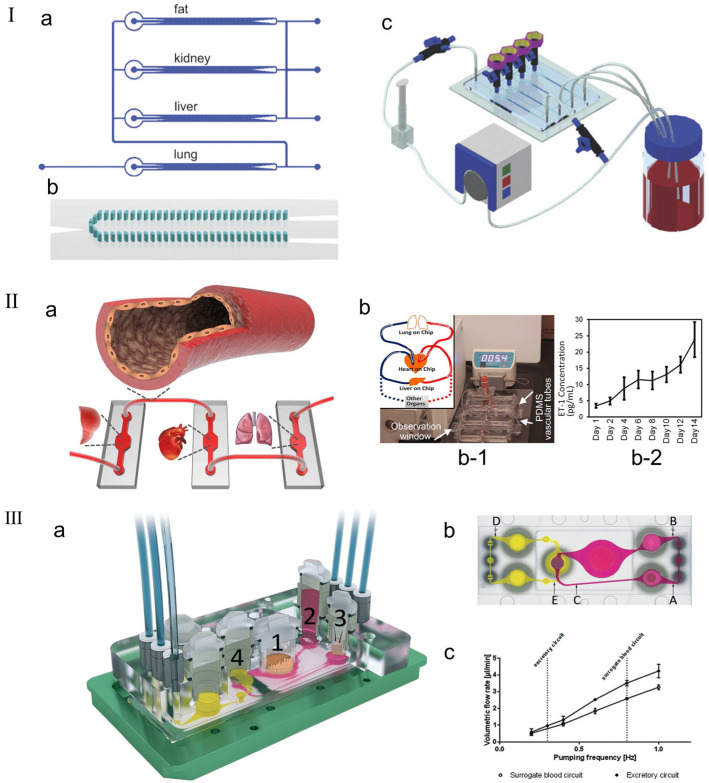
(**I**) Perfusion system setup. (**a**) Schematic diagram of multi-channel 3D-μFCCS. (**b**) Geometry of 3D-μFCCS (micropillar array separates two side channels from the central compartment). (**c**) Closed-loop perfusion culture of cells. During the culture process, the A549 (lung) channel was closed to allow continuous perfusion of the culture medium. Reproduced with permission from [145]. (**II**) (**a**) Schematic diagram of the concept of elastic endothelial blood vessels used to connect multiple organs on a chip system, such as liver, heart and lung modules. (**b**) Endothelialized PDMS vessels for interconnection of multiple organ modules. (**b-1**) The picture shows the assembly of the integrated MOC platform, including the medium reservoir, PDMS vascular tubes, three bioreactors that simulate the organ-on-chip modules, and a flowmeter, in which the medium is circulated using a peristaltic pump. (**b-2**) ETn1 secretion by the HUVECs in the PDMS tubes connecting multiple mimic organ modules. Reproduced with permission from [146]. (**III**) Schematic diagram of the microfluidic four-organ chip device. (**a**) Three-dimensional view of the device, with numbers indicating four tissue culture chambers for intestinal (1), liver (2), skin (3), and kidney (4) tissues. Each tissue culture chamber is depicted in a central cross-section along an array of interconnected microchannels. (**b**) Top view of the four-organ chip layout, showing the locations of three measurement points in the surrogate blood circuit (A, B, and C) and two measurement points in the excretory circuit (D, E). (**c**) Average volume flow rate plotted against pumping frequency for replacement and excretion circulation [147].

In order to simulate and predict the human body’s response to drugs, integrating multiple organ units onto the same platform is a key step in the human chip platform. Existing research groups have reported that they can fuse two, three, or four organ units into a microfluidic system, which share a covering medium and use soluble signals for cell-to-cell communication [141,142,143,144]. Zhang et al. [145] made a three-dimensional microfluidic cell culture platform on a single chip with four compartments for liver, lung, kidney, and adipose tissue culture, indicating that different tissues can indeed communicate through soluble signals (as shown in Figure 10I). Zhang et al. [146] reported the development of a novel vascular module based on PDMS hollow tubes that closely mimics the morphology and properties of human blood vessels to integrate multiple organ chips. Unlike most existing vascular chips or passive tube connections, the reported development involves a simple form of elastic bionic blood vessel that closely mimics the physiological and anatomical properties of blood vessels in the body and can integrate multiple organs on a chip (as shown in the Figure 10II). Li et al. [141] proposed a new multilayer OoC device that can simultaneously evaluate drug metabolism and its drug efficacy and cytotoxicity in different organ-specific cells. Four cell lines representing liver, tumor (breast cancer and lung cancer), and normal tissue (gastric cancer cells) were cultured separately in partitioned microchambers of the multilayer microdevice. Metabolism-independent drug efficacy and toxicity evaluations were effectively performed simultaneously on different organs, indicating that this in vitro model can be simply and reliably applied to drug testing at the MOC level. Maschmeyer et al. [147] fabricated a MOC capable of hosting four human equivalent organs: (1) a reconstructed human 3D small intestine, (2) a skin biopsy selected for oral and dermal substance absorption, (3) an equivalent 3D liver capable of major substance metabolism, and (4) a kidney proximal tubule compartment supporting metabolite excretion (as shown in the Figure 10III). The experimental results demonstrated robust homeostasis and function of these four organs. The four-organ chip co-culture exhibited barrier integrity, continuous gradient molecular transport, and metabolic activity, making it a perfect platform for further in vitro ADME and repeated dose toxicity testing.

### 3.9. ML-Enhanced OoC Models

**Figure 11:**
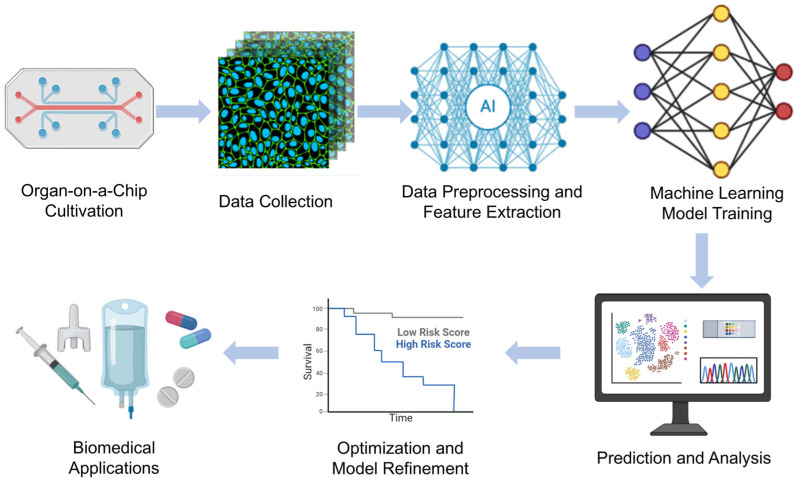
Machine learning-enhanced organ-on-a-chip workflow.

The combination of ML and deep learning with OoC technologies has revolutionized biomedical research by enabling automated analysis, predictive modeling, and real-time decision making. These intelligent approaches aid in data interpretation, optimize experimental design, and enhance the physiological relevance of in vitro models. Below, we highlight recent research examples of ML-enhanced OoC models in various organ systems. Chen et al.’s [23] study mainly introduces a biomimetic lung microphysiological system (Lung-MPS) for studying lung pathology and inflammatory responses. A deep learning algorithm was introduced to analyze and characterize cell activation in this system. This Lung-MPS platform provides an in vitro model that is closer to the real physiological environment for studying COVID-19 and other high-risk infectious lung diseases. Oliver et al. [24] introduced an innovative three-dimensional microfluidic blood–brain microenvironment platform (μmBBN) for culturing and studying the microenvironment of brain metastases. Using AI to identify intrinsic phenotypic differences in cancer cells that can traverse this model can be used to answer basic and translational research questions about metastasis, treatment strategies, and the role of the tumor microenvironment. Shi et al. [25] developed a liver chip system combined with machine learning to improve the safety of drug formulations. The platform, combined with a machine learning algorithm, enables the research team to predict the metabolic behavior and potential toxicity of drugs in the body, thereby optimizing drug formulations and reducing the risk of adverse reactions. Ma et al. [26] developed a heart chip system that combines machine vision and artificial intelligence, aiming to simulate the microenvironment of the human heart to accelerate medical research and drug development. Artificial intelligence algorithms are used to process and interpret large amounts of data acquired from sensors and images, thereby improving the accuracy and efficiency of data analysis. These examples illustrate how machine learning methods are increasingly being integrated with organ-on-a-chip technology to enhance data analysis, optimize experimental conditions, and improve predictive accuracy, ultimately accelerating drug discovery, personalized medicine, and disease modeling in biomedical research. Figure 11 shows the workflow of OoCs combining machine learning (ML) and deep learning (DL), from cell culture to intelligent data analysis to biomedical applications.

## 4. Challenges and Future Perspectives

Currently, OoCs are mainly used in drug development and biomedical research to evaluate the toxicity and efficacy of pharmaceutical candidates and to elucidate disease mechanisms. As precision medicine emerges, OoC technology has set more stringent standards for mimicking human physiology and disease states [148]. The design and fabrication of this technology face challenges related to technical complexity and rising costs, and necessitate the combination of advanced microfluidic technology, innovative materials, and interdisciplinary expertise [149,150]. Furthermore, synergies with artificial intelligence and big data endow OoCs with enhanced data analysis capabilities, thereby facilitating a more precise replication of complex biological phenomena and disease models. During the deployment of this technology, numerous technical obstacles arise, including sustaining cell physiological activity, ensuring cell type diversity, and modeling intricate cell interactions [151,152]. At the same time, the development of OoCs has also raised ethical considerations, particularly regarding the ethical review of human cell usage, with an emphasis on stem cells, and the management and protection of personal biological data. Despite these obstacles, the prospects for OoC technology remain bright. Its utilization in personalized medicine, disease modeling, drug discovery, and toxicity assessments is anticipated to proliferate, offering robust support for biomedical research and novel pharmaceutical development [153,154]. With technological advancements and the intensification of interdisciplinary collaboration, it is anticipated that current technical and ethical challenges will be incrementally surmounted, paving the way for new horizons in medical research and clinical practices, and furthering the goals of precision medicine and personalized therapeutic approaches.

### 4.1. Technical Challenges

**Figure 12:**
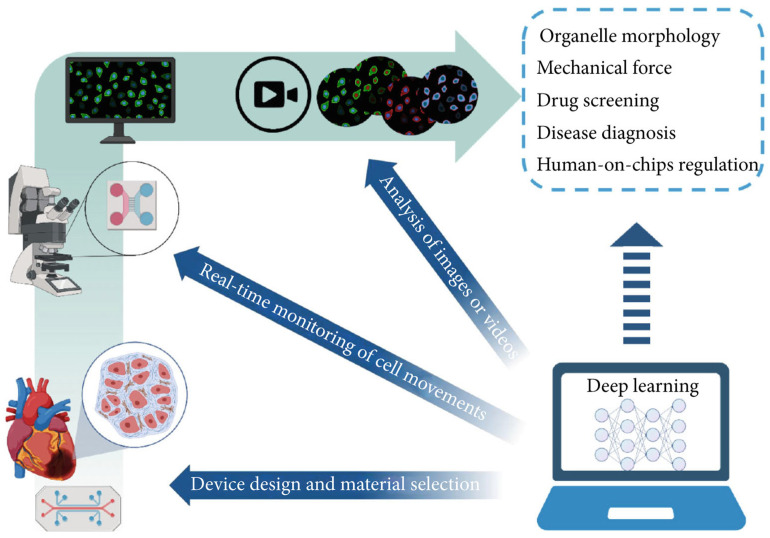
Integration of deep learning with OoCs. Deep learning has been applied to device design, real-time monitoring, and image processing in OoCs. In the future, it may be further applied to organelle tracking, mechanical force mimicking, drug screening, rare disease diagnosis, and human-on-chip regulation. Reproduced with permission from [2].

OoC technology is known for its rapid response capabilities and high-throughput attributes, and has emerged as a significant source of extensive data within the biomedical domain. The considerable parallelization of OoC experiments has resulted in data generation that vastly surpasses the analytical capacity of biomedical researchers via conventional methods [155,156]. This situation necessitates the adoption of automated tools to enhance data processing efficiency and experimental result precision. Deep learning is an important field in machine learning that has achieved remarkable results in various applications such as computer vision, speech recognition, and natural language processing by autonomously identifying and learning patterns in data [157]. Currently, deep learning technology has been integrated into OoC research, finding application in diverse areas including equipment design, real-time monitoring, and image analysis (Figure 12). The fusion of this artificial intelligence technology with OoC systems not only substantially improves the capacity for analyzing complex datasets but also elevates the experimental processes’ level of intelligence [148,150]. This opens up novel avenues for application in fields like high-throughput drug screening, underscoring the immense potential of this technology in future biomedical research and applications.

While significant progress has been made in the development of microfluidic-based OoC technologies, several research gaps and technical challenges remain that hinder their broader application. (1) Lack of Standardized Fabrication Protocols: The absence of standardized manufacturing methods leads to inconsistencies in device design and performance, making cross-laboratory validation and reproducibility difficult. This variation complicates efforts to establish universal benchmarks for OoC systems. (2) Limited Long-term Viability of Cultured Tissues: While OoC systems are effective for short-term experiments, maintaining tissue viability over extended periods remains challenging. This limits their application in chronic disease modeling, where long-term observation is crucial. (3) Integration of Real-Time Data Analytics: Although deep learning and machine learning techniques have been introduced, their integration into OoC platforms for real-time data analysis and predictive modeling is still in its early stages. This limits the ability to conduct dynamic experiments and adjust parameters in real-time based on feedback from the system. (4) Sensor Integration and Data Resolution: Many current sensor technologies struggle with issues related to sensitivity, signal stability, and biocompatibility. Furthermore, ensuring the seamless integration of these sensors within the microfluidic environment without disrupting fluid dynamics or cellular behavior is complex. These limitations hinder the ability to obtain the high-resolution, real-time data necessary for precise modeling and dynamic feedback control in OoC systems.

### 4.2. Ethical Considerations

The development of OoCs represents a quintessentially interdisciplinary endeavor, requiring the collaboration of engineers and biologists to conceive and construct systems that accurately emulate organ-level physiology. As the focus shifts towards personalized medicine and the customization of OoCs, the inclusion of new stakeholders, such as biomedical scientists, clinicians, and patients, becomes imperative in the development trajectory [153]. This integration necessitates the provision of tissue samples and personal health data by patients and physicians, while also demanding attention to ethical considerations including consent, data protection, and ownership. Moreover, this progression obliges the biomedical community to adapt findings from clinical studies into personalized characterizations of OoC models. This adaptation is pivotal for identifying the requisite complexity of cell types, microenvironmental parameters, and tissue interactions. Stakeholders are expected to contribute longitudinal data to aid in validating the health predictions made by the personalized organ chip, thereby enhancing the technology and elucidating its utility in precision medicine [154]. By adopting readout methodologies prevalent in clinical contexts, such as biomarker level assessments and imaging techniques, the functional responses of OoC systems can be directly juxtaposed with actual patient health data. This comparison furnishes avenues for advancements in personalized OoC technology and in the realm of precision medicine, offering significant methodological insights.

### 4.3. Future Directions

Over the next 5–10 years, the development of OoC technologies will be shaped by addressing current limitations and developing emerging innovations. Several key areas are expected to drive this progress: (1) Future OoC platforms will focus on integrating multiple organs (e.g., heart, liver, kidney) to simulate complex inter-organ interactions. This will enable comprehensive modeling of drug absorption, metabolism, and systemic toxicity, supporting personalized medicine and holistic disease understanding. (2) Patient-derived OoCs will allow highly accurate disease models, enabling personalized drug testing, especially in cancer and rare genetic disorders. These platforms will identify the most effective treatments with minimal side effects. (3) The limitations of traditional materials like PDMS—due to poor biocompatibility and mechanical properties—will drive the adoption of novel materials: hydrogels for better cell support and mimicking extracellular matrix properties; elastomeric and stretchable polymers to enhance flexibility and simulate mechanical stimuli. (4) ML and deep learning algorithms will revolutionize OoC platforms, enabling automated data interpretation, predictive modeling, and real-time experimental adjustments. This will significantly enhance drug screening and disease progression analysis. (5) Advances in nanoelectronics and biosensor technologies will allow for precise, real-time data acquisition without compromising microfluidic dynamics.

## 5. Conclusions

The application of microfluidic technology in OoC platforms has emerged as a significant branch of modern biomedical research. By precisely controlling the flow of fluids through microscale channels, microfluidic technology can simulate the microenvironments within the human body, providing cells with more natural growth conditions. This technology enables researchers to construct miniature organ models, such as hearts, livers, and lungs, on chips. These models not only simulate the structure of their respective organs but also their physiological and pathological functions. Through microfluidic technology, accurate drug testing and pathological research can be conducted on these miniature organs. This approach offers a closer approximation to actual human conditions compared to traditional two-dimensional cell cultures and animal models, thus displaying immense potential in drug development, disease model construction, and personalized medicine research. Furthermore, the application of microfluidic technology in OoC platforms has fostered innovation in biomedical research. For instance, in the field of tumor research, researchers can utilize microfluidic technology to construct tumor microenvironment models. These models are capable of simulating the interactions between tumor cells and their surrounding microenvironment, providing an effective platform for investigating tumor growth, metastasis mechanisms, and cancer drug screening. In the realm of personalized medicine, by integrating cells extracted from patients into organ chips, patient-specific pathological states can be simulated, thereby customizing the most suitable treatment plan for each patient.

With continuous advancements and innovations in microfluidic technology, its application in OoC platforms is expected to bring broader prospects to biomedical research and future medical practices. This article systematically introduces the application of microfluidic technology on OoC platforms, emphasizing the critical role of microfluidic technology in replicating the physiological and pathological conditions of human organs, as well as its significance in biomedical and chemical research. Moreover, the contribution of microfluidics in establishing interactive cellular microenvironments that adeptly mimic organ-level tissue architecture and functionality is described, highlighting the utility of this technology in drug screening and toxicity assessment, and its pivotal relevance in personalized medicine. Additionally, it addresses the challenges, ethical considerations, and prospective trajectories within this rapidly evolving field, underscoring the transformative potential of OoC technology in enhancing precision medicine, disease modeling, pharmaceutical development, and toxicity evaluation. Looking ahead, the development of OoC technology is expected to continue its rapid progression, with its applications in precision medicine, disease model construction, drug development, and toxicity evaluation expanding. With technological progress and deeper interdisciplinary research, current technical and ethical challenges are expected to be gradually overcome, further enhancing the fidelity and practicality of the models. Through integration with artificial intelligence, big data analysis, and advanced imaging techniques, OoC platforms are poised to revolutionize our understanding of disease mechanisms, efficient drug screening, and the formulation of personalized treatment strategies, ultimately achieving precise simulation of the complex physiological systems of the human body and opening new chapters in modern medical research and clinical application.

## Figures and Tables

**Table 1:** Comparison of advantages and disadvantages of microfluidic cell culture and other in vitro culture models as well as applicable scenarios.

Culture Model In Vitro	Advantages	Disadvantages	Common Equipment	Scope of Application
Traditional 2D Cell Culture	Simple operation, low cost, suitable for basic research and large-scale screening	Lack of three-dimensional structure and limited physiological relevance	Petri dishes, culture bottles, transosmotic membranes	Basic cell biology, preliminary drug screening, teaching and demonstration
Traditional 3D cell culture	Close to the three-dimensional structure in the body, cell-to-cell interactions, suitable for disease models and tissue engineering	Operation and costs are relatively high, and observation and analysis are more difficult	Engineered culture scaffolds, spheres, microcarriers, tissue biopsies, organoids	Basic cell research cancer research, tissue engineering, disease modeling
Microfluidic 2D/3D cell culture (OoC) (Simulate microenvironmental and dynamic conditions in 2D or 3D cell culture)	Simulation of complex microenvironments, high-precision control, simulation of dynamic conditions for advanced disease models and drug screening	The cost is high, the operation is complex, and the equipment needs are specialized	Microfluidic chips, micropumps and valves, integrated sensors, organ chips	Basic cell research complex disease research, drug development and screening, basic research in biology

**Table 2:** Comparison of different microfluidic chip fabrication methods.

Method	Fabrication Complexity	Time Required	Specialty Equipment Needed	Costs	Application Advantages	Limitations
Soft Lithography [33,34]	Medium	Hours–Days	Photolithography Setup	High	High-resolution features, biocompatibility, widely used	Requires cleanroom, limited scalability
Hot Embossing [36,37]	Medium	Minutes–Hours	Hot Press	Medium	High precision, suitable for commercial-scale production	Limited to thermoplastics, high setup costs
Injection Molding [36,38]	High	Hours–Days	Injection Molding Machine	High	High-throughput, consistent results, scalable	Expensive molds, high initial investment
3D Printing [39,40,41,42,43]	Low–Medium	Minutes–Hours	3D Printer	Low–Medium	Rapid prototyping, customization, cost-efficient	Resolution limitations, material constraints
Laser-cut Synthetic Biomaterials [44,45]	Medium	Hours–Days	Laser Cutter	Medium	Allows controlled microenvironments, supports tissue engineering	Requires specialized materials and patterning
CNC Milling [46]	Medium	Hours	CNC Milling Machine	Medium	Precision machining, allows open-top microfluidics	Limited by tool size, not suitable for high-throughput
Paper-based Microenvironments [47]	Low	Minutes–Hours	Simple Fabrication Tools	Low	Low-cost, adaptable to tumor models, hypoxia research	Limited mechanical strength, hard to scale
Roll-to-Roll Manufacturing [48]	Medium	Hours	Continuous Printing System	Medium	High-speed production, scalable for flexible substrates	Requires roll-compatible substrates, material restrictions
Flexography [49]	Low	Minutes–Hours	Printing Plates	Low	Fast pattern transfer, ideal for polymer molds	Not suitable for high-precision structures
Microthermoforming [50]	Medium	Hours	Heating Press	Medium	Allows suspended microstructures, suitable for 3D designs	Limited to thermoplastics, requires heating setup
Focused Ion Beam Milling [51]	High	Hours–Days	Focused Ion Beam System	High	Nano-scale precision, advanced material compatibility	High cost, limited scalability
Laser Micromachining [52]	High	Minutes–Hours	High-Precision Laser System	High	Rapid prototyping, supports complex structures	Equipment-intensive, high energy consumption

**Table 3:** Comparison and application overview of different types of OoC technologies.

OoC Type	Structural Features	Key Materials	Applications	Challenges and Limitations	ML/DL Integration
Lung-on-a-Chip [63,64,65,66,67,68,69,70,71,72,73,74,75,76,77,78,79]	Mimics alveolar–capillary interface, airflow, and mechanical stretching	PDMS, PMMA, hydrogel-based membranes	Pulmonary drug screening, respiratory disease modeling, COVID-19 research	Limited alveolar network complexity, synthetic membranes differ from ECM	Deep learning models for COVID-19 lung infection research
Liver-on-a-Chip [80,81,82,83,84,85,86,87]	Recreates liver lobules, bile ducts, and metabolic zones	GelMA, decellularized liver matrix, PDMS	Hepatotoxicity assessment, metabolic disease modeling, liver regeneration studies	Difficult to replicate full liver zonation and bile secretion processes	ML for drug metabolism and hepatotoxicity prediction
Heart-on-a-Chip [88,89,90,91,92,93,94,95,96,97,98]	Models myocardial contractility, electrical conductivity, and fluid dynamics	PDMS, elastomers, 3D-bioprinted cardiac tissues	Cardiotoxicity screening, heart disease modeling, drug testing	Mechanical stress does not fully mimic in vivo cardiac conditions	AI-enhanced cardiac tissue analysis and contractility monitoring
Gut-on-a-Chip [99,100,101,102,103,104,105,106,107,108,109,110]	Replicates gut peristalsis, microbiota interaction, and epithelial barrier	PDMS, extracellular matrix proteins, TEER electrodes	Intestinal permeability studies, microbiome research, IBD modeling	Gut immune and endocrine responses not fully replicated	Machine learning for gut barrier integrity and microbiome interactions
Brain-on-a-Chip [111,112,113,114,115,116]	Simulates blood–brain barrier, neural networks, and brain microenvironment	Hydrogel scaffolds, PDMS, biomimetic brain tissues	Neurodegenerative disease modeling, blood–brain barrier permeability testing	Complex neural interactions and long-term stability remain challenging	AI-assisted neurodegenerative disease progression modeling
Kidney-on-a-Chip [117,118,119,120,121,122]	Replicates glomerular filtration, tubular reabsorption, and nephrotoxicity screening	PDMS, synthetic basement membranes, ECM proteins	Renal toxicity assessment, nephropathy modeling, drug screening	Limited ability to model chronic kidney disease progression	DL-based nephrotoxicity screening and renal function assessment
Cancer-on-a-Chip [123,124,125,126,127,128,129,130,131,132]	Emulates tumor microenvironment, angiogenesis, and metastasis	Hydrogels, 3D-printed tumor structures, PMMA	Cancer drug testing, tumor metastasis studies, precision oncology	Heterogeneity of tumor models, difficulty in replicating immune responses	AI-driven cancer cell tracking, drug response prediction, and treatment optimization

## References

[1] Schimek K., Busek M., Brincker S., Groth B., Hoffmann S., Lauster R., Lindner G., Lorenz A., Menzel U., Sonntag F. (2013). Integrating biological vasculature into a multi-organ-chip microsystem. Lab Chip.

[2] Li J., Chen J., Bai H., Wang H., Hao S., Ding Y., Peng B., Zhang J., Li L., Huang W. (2022). An Overview of Organs-on-Chips Based on Deep Learning. Research.

[3] Park T.E., Mustafaoglu N., Herland A., Hasselkus R., Mannix R., FitzGerald E.A., Prantil-Baun R., Watters A., Henry O., Benz M. (2019). Hypox-ia-enhanced Blood-Brain Barrier Chip recapitulates human barrier function and shuttling of drugs and antibodies. Nat. Commun..

[4] Si L., Bai H., Rodas M., Cao W., Oh C.Y., Jiang A., Moller R., Hoagland D., Oishi K., Horiuchi S. (2021). A human-airway-on-a-chip for the rapid identification of candidate antiviral therapeutics and prophylactics. Nat. Biomed. Eng..

[5] Seok J., Warren H.S., Cuenca A.G., Mindrinos M.N., Baker H.V., Xu W., Richards D.R., McDonald-Smith G.P., Gao H., Hennessy L. (2013). Genomic responses in mouse models poorly mimic human inflammatory diseases. Proc. Natl. Acad. Sci. USA.

[6] Nguyen H.-T., Rissanen S.-L., Peltokangas M., Laakkonen T., Kettunen J., Barthod L., Sivakumar R., Palojärvi A., Junttila P., Talvitie J. (2024). Highly scalable and standardized organ-on-chip platform with TEER for biological barrier modeling. Tissue Barriers.

[7] Sønstevold L., Koza P., Czerkies M., Andreassen E., McMahon P., Vereshchagina E. (2024). Prototyping in Polymethylpentene to Enable Oxygen-Permeable On-a-Chip Cell Culture and Organ-on-a-Chip Devices Suitable for Microscopy. Micromachines.

[8] Azizgolshani H., Coppeta J.R., Vedula E.M., Marr E.E., Cain B.P., Luu R.J., Lech M.P., Kann S.H., Mulhern T.J., Tandon V. (2021). High-throughput organ-on-chip platform with integrated programmable fluid flow and real-time sensing for complex tissue models in drug development workflows. Lab Chip.

[9] Kann S.H., Shaughnessey E.M., Coppeta J.R., Azizgolshani H., Isenberg B.C., Vedula E.M., Zhang X., Charest J.L. (2022). Measurement of oxygen consumption rates of human renal proximal tubule cells in an array of organ-on-chip devices to monitor drug-induced metabolic shifts. Microsyst. Nanoeng..

[10] Wang Y.I., Shuler M.L. (2018). UniChip enables long-term recirculating unidirectional perfusion with gravity-driven flow for microphysiological systems. Lab A Chip.

[11] Strelez C., Perez R., Chlystek J.S., Cherry C., Yoon A.Y., Haliday B., Shah C., Ghaffarian K., Sun R.X., Jiang H. (2023). Integration of Patient-Derived Organoids and Organ-on-Chip Systems: Investigating Colorectal Cancer Invasion within the Mechanical and GABAergic Tumor Microenvironment. bioRxiv.

[12] Moya A., Ortega-Ribera M., Guimerà X., Sowade E., Zea M., Illa X., Ramon E., Villa R., Gracia-Sancho J., Gabriel G. (2018). Online oxygen monitoring using integrated inkjet-printed sensors in a liver-on-a-chip system. Lab Chip.

[13] Yang Y., Gao X., Widdicombe B., Zhang X., Zielinski J.L., Cheng T., Gunatilaka A., Leung K.K., Plaxco K.W., Unnithan R.R. (2024). Dual-Purpose Aptamer-Based Sensors for Real-Time, Multiplexable Monitoring of Metabolites in Cell Culture Media. ACS Nano.

[14] Ingber D.E. (2018). Developmentally inspired human ‘organs on chips’. Development.

[15] Gadde M., Phillips C., Ghousifam N., Sorace A.G., Wong E., Krishnamurthy S., Syed A., Rahal O., Yankeelov T.E., Woodward W.A. (2020). In vitro vascularized tumor platform for modeling tumor-vasculature interactions of inflammatory breast cancer. Biotechnol. Bioeng..

[16] Guimaraes A.P.P., Calori I.R., Stilhano R.S., Tedesco A.C. (2024). Renal proximal tubule-on-a-chip in PDMS: Fabrication, functionalization, and RPTEC:HUVEC co-culture evaluation. Biofabrication.

[17] Lee J.-B., Kim H., Kim S., Sung G.Y. (2022). Fabrication and Evaluation of Tubule-on-a-Chip with RPTEC/HUVEC Co-Culture Using Injection-Molded Polycarbonate Chips. Micromachines.

[18] Borenstein J.T., Vunjak-Novakovic G. (2011). Engineering Tissue with BioMEMS. IEEE Pulse.

[19] Bhatia S.N., Ingber D.E. (2014). Microfluidic organs-on-chips. Nat. Biotechnol..

[20] Gizzi A., Giannitelli S.M., Trombetta M., Cherubini C., Filippi S., De Ninno A., Businaro L., Gerardino A., Rainer A. (2017). Computationally Informed Design of a Multi-Axial Actuated Microfluidic Chip Device. Sci. Rep..

[21] Prevedello L., Michielin F., Balcon M., Savio E., Pavan P., Elvassore N. (2018). A Novel Microfluidic Platform for Biomechano-Stimulations on a Chip. Ann. Biomed. Eng..

[22] Nof E., Zidan H., Artzy-Schnirman A., Mouhadeb O., Beckerman M., Bhardwaj S., Elias-Kirma S., Gur D., Beth-Din A., Levenberg S. (2022). Human Multi-Compartment Airways-on-Chip Platform for Emulating Respiratory Airborne Transmission: From Nose to Pulmonary Acini. Front. Physiol..

[23] Chen Z., Huang J., Zhang J., Xu Z., Li Q., Ouyang J., Yan Y., Sun S., Ye H., Wang F. (2022). A storm in a teacup—A biomimetic lung microphysiological system in conjunction with a deep-learning algorithm to monitor lung pathological and inflammatory reactions. Biosens. Bioelectron..

[24] Oliver C.R., Westerhof T.M., Castro M.G., Merajver S.D. (2020). Quantifying the Brain Metastatic Tumor Micro-Environment using an Organ-On-A Chip 3D Model, Machine Learning, and Confocal Tomography. J. Vis. Exp..

[25] Shi Y., Lin C.H., Reker D., Steiger C., Hess K., Collins J.E., Tamang S., Ishida K., Lopes A., Wainer J. (2022). A machine learning liver-on-a-chip system for safer drug formulation. bioRxiv.

[26] Ma X., Barros N., Zhao J. (2024). Heart-on-a-chip based on machine vision and artificial intelligence. Authorea.

[27] Hu T., Han W., Zhou Y., Tu W., Li X., Ni Z. (2024). Flow-electricity Coupling Fields Enhanced Microfluidic Platforms for Efficient Exosomes Isolation. Anal. Methods.

[28] Au S.H., Chamberlain M.D., Mahesh S., Sefton M.V., Wheeler A.R. (2014). Hepatic organoids for microfluidic drug screening. Lab Chip.

[29] Bhattacharjee N., Urrios A., Kang S., Folch A. (2016). The upcoming 3D-printing revolution in microfluidics. Lab Chip.

[30] Simpson C.R., Kelly H.M., Murphy C.M. (2020). Synergistic use of biomaterials and licensed therapeutics to manipulate bone remodelling and promote non-union fracture repair. Adv. Drug Deliv. Rev..

[31] Faley S., Seale K., Hughey J., Schaffer D.K., VanCompernolle S., McKinney B., Baudenbacher F., Unutmaz D., Wikswo J.P. (2008). Microfluidic platform for real-time signaling analysis of multiple single T cells in parallel. Lab Chip.

[32] Lu R.X.Z., Radisic M. (2021). Organ-on-a-chip platforms for evaluation of environmental nanoparticle toxicity. Bioact. Mater..

[33] Amadeo F., Mukherjee P., Gao H., Zhou J., Papautsky I. (2021). Polycarbonate Masters for Soft Lithography. Micromachines.

[34] Xia Y., Whitesides G.M. (1998). Soft Lithography. Angew. Chem. (Int. Ed. Engl.).

[35] Tam R.Y., Smith L.J., Shoichet M.S. (2017). Engineering Cellular Microenvironments with Photo- and Enzymatically Responsive Hydrogels: Toward Biomimetic 3D Cell Culture Models. Acc. Chem. Res..

[36] Scott S.M., Ali Z. (2021). Fabrication Methods for Microfluidic Devices: An Overview. Micromachines.

[37] Bhandari A., Khatri N., Mishra Y.K., Goyat M.S. (2023). Superhydrophobic coatings by the hot embossing approach: Recent developments and state-of-art applications. Materials Today Chemistry.

[38] Szydzik C., Gavela A.F., Herranz S., Roccisano J., Knoerzer M., Thurgood P., Khoshmanesh K., Mitchell A., Lechuga L.M. (2017). An automated optofluidic biosensor platform combining interferometric sensors and injection moulded microfluidics. Lab Chip.

[39] De Almeida Monteiro Melo Ferraz M., Nagashima J.B., Venzac B., Le Gac S., Songsasen N. (2020). 3D printed mold leachates in PDMS microfluidic devices. Sci. Rep..

[40] Comina G., Suska A., Filippini D. (2014). Low cost lab-on-a-chip prototyping with a consumer grade 3D printer. Lab Chip.

[41] Rusling J.F. (2018). Developing Microfluidic Sensing Devices Using 3D Printing. ACS Sens..

[42] Gowers S.A.N., Curto V.F., Seneci C.A., Wang C., Anastasova S., Vadgama P., Yang G.-Z., Boutelle M.G. (2015). 3D Printed Microfluidic Device with Integrated Biosensors for Online Analysis of Subcutaneous Human Microdialysate. Anal. Chem..

[43] Bishop G.W., Satterwhite-Warden J.E., Bist I., Chen E., Rusling J.F. (2015). Electrochemiluminescence at Bare and DNA-Coated Graphite Electrodes in 3D-Printed Fluidic Devices. ACS Sens..

[44] Gjorevski N., Nikolaev M., Brown T.E., Mitrofanova O., Brandenberg N., DelRio F.W., Yavitt F.M., Liberali P., Anseth K.S., Lutolf M.P. (2022). Tissue geometry drives deterministic organoid patterning. Science.

[45] Manfrin A., Tabata Y., Paquet E.R., Vuaridel A.R., Rivest F.R., Naef F., Lutolf M.P. (2019). Engineered signaling centers for the spatially controlled patterning of human pluripotent stem cells. Nat. Methods.

[46] Yu J., Berthier E., Craig A., de Groot T.E., Sparks S., Ingram P.N., Jarrard D.F., Huang W., Beebe D.J., Theberge A.B. (2019). Reconfigurable open microfluidics for studying the spatiotemporal dynamics of paracrine signalling. Nat. Biomed. Eng..

[47] Rodenhizer D., Gaude E., Cojocari D., Mahadevan R., Frezza C., Wouters B., McGuigan A.P. (2016). A three-dimensional engineered tumour for spatial snapshot analysis of cell metabolism and phenotype in hypoxic gradients. Nat. Mater..

[48] Tahir U., Kim J.I., Javeed S., Khaliq A., Kim J.-H., Kim D.-I., Jeong M.Y. (2022). Process Optimization for Manufacturing Functional Nanosurfaces by Roll-to-Roll Nanoimprint Lithography. Nanomaterials.

[49] Olmos C.M., Vaca A., Rosero G., Peñaherrera A., Perez C., Carneiro I.d.S., Vizuete K., Arroyo C.R., Debut A., Pérez M.S. (2019). Epoxy resin mold and PDMS microfluidic devices through photopolymer flexographic printing plate. Sens. Actuators B: Chem..

[50] Truckenmüller R., Giselbrecht S., van Blitterswijk C., Dambrowsky N., Gottwald E., Mappes T., Rolletschek A., Saile V., Trautmann C., Weibezahn K.-F. (2008). Flexible fluidic microchips based on thermoformed and locally modified thin polymer films. Lab Chip.

[51] Lebedev D., Malyshev G., Ryzhkov I., Mozharov A., Shugurov K., Sharov V., Panov M., Tumkin I., Afonicheva P., Evstrapov A. (2021). Focused ion beam milling based formation of nanochannels in silicon-glass microfluidic chips for the study of ion transport. Microfluid. Nanofluidics.

[52] Konari P.R., Clayton Y.-D., Vaughan M.B., Khandaker M., Hossan M.R. (2021). Experimental Analysis of Laser Micromachining of Microchannels in Common Microfluidic Substrates. Micromachines.

[53] Reardon S. (2015). ‘Organs-on-chips’ go mainstream. Nature.

[54] Kim D., Wu X., Young A.T., Haynes C.L. (2014). Microfluidics-Based in Vivo Mimetic Systems for the Study of Cellular Biology. Acc. Chem. Res..

[55] Huh D., Kim H.J., Fraser J.P., Shea D.E., Khan M., Bahinski A., Hamilton G.A., Ingber D.E. (2013). Microfabrication of human organs-on-chips. Nat. Protoc..

[56] Kim H.J., Li H., Collins J.J., Ingber D.E. (2015). Contributions of microbiome and mechanical deformation to intestinal bacterial overgrowth and inflammation in a human gut-on-a-chip. Proc. Natl. Acad. Sci. USA.

[57] Varone A., Nguyen J.K., Leng L., Barrile R., Sliz J., Lucchesi C., Wen N., Gravanis A., Hamilton G.A., Karalis K. (2021). A novel organ-chip system emulates three-dimensional architecture of the human epithelia and the mechanical forces acting on it. Biomaterials.

[58] Shin W., Kim H.J. (2018). Pathomimetic modeling of human intestinal diseases and underlying host-gut microbiome interactions in a gut-on-a-chip. Methods Cell Biol..

[59] Kim H.J., Lee J., Choi J.-H., Bahinski A., Ingber D.E. (2016). Co-culture of Living Microbiome with Microengineered Human Intestinal Villi in a Gut-on-a-Chip Microfluidic Device. J. Vis. Exp..

[60] Kim H.J., Ingber D.E. (2013). Gut-on-a-Chip microenvironment induces human intestinal cells to undergo villus differentiation. Integr. Biol..

[61] Kim H.J., Huh D., Hamilton G., Ingber D.E. (2012). Human gut-on-a-chip inhabited by microbial flora that experiences intestinal peristalsis-like motions and flow. Lab Chip.

[62] Douville N.J., Zamankhan P., Tung Y.-C., Li R., Vaughan B.L., Tai C.-F., White J., Christensen P.J., Grotberg J.B., Takayama S. (2010). Combination of fluid and solid mechanical stresses contribute to cell death and detachment in a microfluidic alveolar model. Lab Chip.

[63] Benam K.H., Mazur M., Choe Y., Ferrante T.C., Novak R., Ingber D.E. (2017). Human Lung Small Airway-on-a-Chip Protocol. Methods Mol. Biol..

[64] Humayun M., Chow C.-W., Young E.W.K. (2018). Microfluidic lung airway-on-a-chip with arrayable suspended gels for studying epithelial and smooth muscle cell interactions. Lab Chip.

[65] Benam K.H., Villenave R., Lucchesi C., Varone A., Hubeau C., Lee H.-H., E Alves S., Salmon M., Ferrante T.C., Weaver J.C. (2015). Small airway-on-a-chip enables analysis of human lung inflammation and drug responses in vitro. Nat. Methods.

[66] Sun A.M., Hoffman T., Luu B.Q., Ashammakhi N., Li S. (2021). Application of lung microphysiological systems to COVID-19 modeling and drug discovery: A review. Bio-Des. Manuf..

[67] Kim W., Lee Y., Kang D., Kwak T., Lee H.-R., Jung S. (2023). 3D Inkjet-Bioprinted Lung-on-a-Chip. ACS Biomater. Sci. Eng..

[68] Zhang M., Xu C., Jiang L., Qin J. (2018). A 3D human lung-on-a-chip model for nanotoxicity testing. Toxicol. Res..

[69] Tavana H., Kuo C.-H., Lee Q.Y., Mosadegh B., Huh D., Christensen P.J., Grotberg J.B., Takayama S. (2009). Dynamics of Liquid Plugs of Buffer and Surfactant Solutions in a Micro-Engineered Pulmonary Airway Model. Langmuir.

[70] Xu C., Zhang M., Chen W., Jiang L., Chen C., Qin J. (2020). Assessment of Air Pollutant PM2.5 Pulmonary Exposure Using a 3D Lung-on-Chip Model. ACS Biomater. Sci. Eng..

[71] Sengupta A., Roldan N., Kiener M., Froment L., Raggi G., Imler T., de Maddalena L., Rapet A., May T., Carius P. (2022). A New Immortalized Human Alveolar Epithelial Cell Model to Study Lung Injury and Toxicity on a Breathing Lung-On-Chip System. Front. Toxicol..

[72] Sengupta A., Dorn A., Jamshidi M., Schwob M., Hassan W., De Maddalena L.L., Hugi A., Stucki A.O., Dorn P., Marti T.M. (2023). A multiplex inhalation platform to model in situ like aerosol delivery in a breathing lung-on-chip. Front. Pharmacol..

[73] van Os L., Yeoh J., Witz G., Ferrari D., Krebs P., Chandorkar Y., Zeinali S., Sengupta A., Guenat O.T. (2023). Immune cell extravasation in an organ-on-chip to model lung inflammation. Eur. J. Pharm. Sci..

[74] Huh D., Matthews B.D., Mammoto A., Montoya-Zavala M., Hsin H.Y., Ingber D.E. (2010). Reconstituting Organ-Level Lung Functions on a Chip. Science.

[75] Zamprogno P., Wüthrich S., Achenbach S., Thoma G., Stucki J.D., Hobi N., Schneider-Daum N., Lehr C.-M., Huwer H., Geiser T. (2021). Second-generation lung-on-a-chip with an array of stretchable alveoli made with a biological membrane. Commun. Biol..

[76] Shen C., Yang H., She W., Meng Q. (2023). A microfluidic lung-on-a-chip based on biomimetic hydrogel membrane. Biotechnol. Bioeng..

[77] Huang D., Liu T., Liao J., Maharjan S., Xie X., Pérez M., Anaya I., Wang S., Mayer A.T., Kang Z. (2021). Reversed-engineered human alveolar lung-on-a-chip model. Proc. Natl. Acad. Sci. USA.

[78] You Y., Zhang C., Guo Z., Xu F., Sun D., Xia J., Chen S. (2024). Lung-on-a-chip composed of styrene-butadiene-styrene nano-fiber/porous PDMS composite membranes with cyclic triaxial stimulation. Microfluid. Nanofluidics.

[79] She W., Shen C., Xue Z., Zhang B., Zhang G., Meng Q. (2025). Hydrogel Strain Sensors for Integrating Into Dynamic Organ-on-a-Chip. Small.

[80] Foster A.J., Chouhan B., Regan S.L., Rollison H., Amberntsson S., Andersson L.C., Srivastava A., Darnell M., Cairns J., Lazic S.E. (2019). Integrated in vitro models for hepatic safety and metabolism: Evaluation of a human Liver-Chip and liver spheroid. Arch. Toxicol..

[81] A Banaeiyan A., Theobald J., Paukštyte J., Wölfl S., Adiels C.B., Goksör M. (2017). Design and fabrication of a scalable liver-lobule-on-a-chip microphysiological platform. Biofabrication.

[82] Xie X., Maharjan S., Kelly C., Liu T., Lang R.J., Alperin R., Sebastian S., Bonilla D., Gandolfo S., Boukataya Y. (2021). Customizable Microfluidic Origami Liver-on-a-Chip (oLOC). Adv. Mater. Technol..

[83] Wang E., Andrade M.J., Smith Q. (2023). Vascularized liver-on-a-chip model to investigate nicotine-induced dysfunction. Biomicrofluidics.

[84] Jiao D., Xie L., Xing W. (2024). A pumpless liver-on-a-chip for drug hepatotoxicity analysis. Analyst.

[85] Yang J., Khorsandi D., Trabucco L., Ahmed M., Khademhosseini A., Dokmeci M.R., Ye J.Y., Jucaud V. (2024). Liver-on-a-Chip Integrated with Label-Free Optical Biosensors for Rapid and Continuous Monitoring of Drug-Induced Toxicity. Small.

[86] Ya S., Ding W., Li S., Du K., Zhang Y., Li C., Liu J., Li F., Li P., Luo T. (2021). On-Chip Construction of Liver Lobules with Self-Assembled Perfusable Hepatic Sinusoid Networks. ACS Appl. Mater. Interfaces.

[87] Lu S., Cuzzucoli F., Jiang J., Liang L.-G., Wang Y., Kong M., Zhao X., Cui W., Li J., Wang S. (2018). Development of a biomimetic liver tumor-on-a-chip model based on decellularized liver matrix for toxicity testing. Lab Chip.

[88] Landau S., Zhao Y., Hamidzada H., Kent G.M., Okhovatian S., Lu R.X.Z., Liu C., Wagner K.T., Cheung K., Shawky S.A. (2024). Primitive macrophages enable long-term vascularization of human heart-on-a-chip platforms. Cell Stem Cell.

[89] Jayne R.K., Karakan M.Ç., Zhang K., Pierce N., Michas C., Bishop D.J., Chen C.S., Ekinci K.L., White A.E. (2021). Direct laser writing for cardiac tissue engineering: A microfluidic heart on a chip with integrated transducers. Lab Chip.

[90] Bannerman D., Pascual-Gil S., Wu Q., Fernandes I., Zhao Y., Wagner K.T., Okhovatian S., Landau S., Rafatian N., Bodenstein D.F. (2024). Heart-on-a-Chip Model of Epicardial–Myocardial Interaction in Ischemia Reperfusion Injury. Adv. Health Mater..

[91] Thavandiran N., Dubois N., Mikryukov A., Massé S., Beca B., Simmons C.A., Deshpande V.S., McGarry J.P., Chen C.S., Nanthakumar K. (2013). Design and formulation of functional pluripotent stem cell-derived cardiac microtissues. Proc. Natl. Acad. Sci. USA.

[92] Sidorov V.Y., Samson P.C., Sidorova T.N., Davidson J.M., Lim C.C., Wikswo J.P. (2017). I-Wire Heart-on-a-Chip I: Three-dimensional cardiac tissue constructs for physiology and pharmacology. Acta Biomater..

[93] Lind J.U., Busbee T.A., Valentine A.D., Pasqualini F.S., Yuan H., Yadid M., Park S.-J., Kotikian A., Nesmith A.P., Campbell P.H. (2016). Instrumented cardiac microphysiological devices via multimaterial three-dimensional printing. Nat. Mater..

[94] Kieda J., Shakeri A., Landau S., Wang E.Y., Zhao Y., Lai B.F., Okhovatian S., Wang Y., Jiang R., Radisic M. (2023). Advances in cardiac tissue engineering and heart-on-a-chip. J. Biomed. Mater. Res. Part A.

[95] Schroer A.K., Shotwell M.S., Sidorov V.Y., Wikswo J.P., Merryman W.D. (2017). I-Wire Heart-on-a-Chip II: Biomechanical analysis of contractile, three-dimensional cardiomyocyte tissue constructs. Acta Biomater..

[96] Zhao Y., Rafatian N., Wang E.Y., Wu Q., Lai B.F., Lu R.X., Savoji H., Radisic M. (2020). Towards chamber specific heart-on-a-chip for drug testing applications. Adv. Drug Deliv. Rev..

[97] Liu Y., Liu Y., Kamran R., Kamran R., Han X., Han X., Wang M., Wang M., Li Q., Li Q. (2024). Human heart-on-a-chip microphysiological system comprising endothelial cells, fibroblasts, and iPSC-derived cardiomyocytes. Sci. Rep..

[98] Mourad O., Yee R., Li M., Nunes S.S. (2023). Modeling Heart Diseases on a Chip: Advantages and Future Opportunities. Circ. Res..

[99] Sedrani C., Gomez-Giro G., Grandmougin L., Schwamborn J.C., Wilmes P. (2023). A Gut-on-a-Chip Model to Study the Gut Microbiome-Nervous System Axis. J. Vis. Exp..

[100] Han J., Wang Y., Ding J., Chen H., Shi C., Li X., Xu Z., Chen J., Kong F., Wang L. (2024). Gut-on-a-Chip Reveals Enhanced Peristalsis Reduces Nanoplastic-Induced Inflammation. Small.

[101] Wang L., Han J., Su W., Li A., Zhang W., Li H., Hu H., Song W., Xu C., Chen J. (2023). Gut-on-a-chip for exploring the transport mechanism of Hg(II). Microsyst. Nanoeng..

[102] Jeon M.S., Choi Y.Y., Mo S.J., Ha J.H., Lee Y.S., Lee H.U., Park S.D., Shim J.-J., Lee J.-L., Chung B.G. (2022). Contributions of the microbiome to intestinal inflammation in a gut-on-a-chip. Nano Converg..

[103] Renous N., Kiri M.D., Barnea R.A., Rauti R., Leichtmann-Bardoogo Y., Maoz B.M. (2021). Spatial trans-epithelial electrical resistance (S-TEER) integrated in organs-on-chips. Lab Chip.

[104] Thomas D.P., Zhang J., Nguyen N.T., Ta H.T. (2023). Microfluidic Gut-on-a-Chip: Fundamentals and Challenges. Biosensors.

[105] Odijk M., van der Meer A.D., Levner D., Kim H.J., van der Helm M.W., Segerink L.I., Frimat J.-P., Hamilton G.A., Ingber D.E., Berg A.v.D. (2014). Measuring direct current trans-epithelial electrical resistance in organ-on-a-chip microsystems. Lab Chip.

[106] van der Helm M.W., Odijk M., Frimat J.-P., van der Meer A.D., Eijkel J.C., Berg A.v.D., Segerink L.I. (2017). Fabrication and Validation of an Organ-on-chip System with Integrated Electrodes to Directly Quantify Transendothelial Electrical Resistance. J. Vis. Exp..

[107] Shin Y.C., Than N., Park S.J., Kim H.J. (2024). Bioengineered human gut-on-a-chip for advancing non-clinical pharmaco-toxicology. Expert Opin. Drug Metab. Toxicol..

[108] van der Helm M.W., Odijk M., Frimat J.-P., van der Meer A.D., Eijkel J.C., Berg A.v.D., Segerink L.I. (2016). Direct quantification of transendothelial electrical resistance in organs-on-chips. Biosens. Bioelectron..

[109] Lucchetti M., Werr G., Johansson S., Barbe L., Grandmougin L., Wilmes P., Tenje M. (2024). Integration of multiple flexible electrodes for real-time detection of barrier formation with spatial resolution in a gut-on-chip system. Microsyst. Nanoeng..

[110] Vera D., García-Díaz M., Torras N., Castillo Ó., Illa X., Villa R., Alvarez M., Martinez E. (2024). A 3D bioprinted hydrogel gut-on-chip with integrated electrodes for transepithelial electrical resistance (TEER) measurements. Biofabrication.

[111] Maoz B.M. (2021). Brain-on-a-Chip: Characterizing the next generation of advanced in vitro platforms for modeling the central nervous system. APL Bioeng..

[112] Amirifar L., Shamloo A., Nasiri R., de Barros N.R., Wang Z.Z., Unluturk B.D., Libanori A., Ievglevskyi O., Diltemiz S.E., Sances S. (2022). Brain-on-a-chip: Recent advances in design and techniques for microfluidic models of the brain in health and disease. Biomaterials.

[113] Chim S.M., Howell K., Kokkosis A., Zambrowicz B., Karalis K., Pavlopoulos E. (2024). A Human Brain-Chip for Modeling Brain Pathologies and Screening Blood–Brain Barrier Crossing Therapeutic Strategies. Pharmaceutics.

[114] Brofiga M., Massobrio P. (2022). Brain-on-a-Chip: Dream or Reality?. Front. Neurosci..

[115] Herland A., van der Meer A.D., FitzGerald E.A., Park T.-E., Sleeboom J.J.F., Ingber D.E. (2016). Distinct Contributions of Astrocytes and Pericytes to Neuroinflammation Identified in a 3D Human Blood-Brain Barrier on a Chip. PLoS ONE.

[116] Choi J.-W., Seo M., Kim K., Kim A.-R., Lee H., Kim H.-S., Park C.G., Cho S.W., Kang J.H., Joo J. (2023). Aptamer Nanoconstructs Crossing Human Blood–Brain Barrier Discovered via Microphysiological System-Based SELEX Technology. ACS Nano.

[117] Sakolish C.M., Philip B., Mahler G.J. (2019). A human proximal tubule-on-a-chip to study renal disease and toxicity. Biomicrofluidics.

[118] Jang K.-J., Mehr A.P., Hamilton G.A., McPartlin L.A., Chung S., Suh K.-Y., Ingber D.E. (2013). Human kidney proximal tubule-on-a-chip for drug transport and nephrotoxicity assessment. Integr. Biol..

[119] Kim K., Jeong B., Lee Y.-M., Son H.-E., Ryu J.-Y., Park S., Jeong J.C., Chin H.J., Kim S. (2022). Three-Dimensional Kidney-on-a-Chip Assessment of Contrast-Induced Kidney Injury: Osmolality and Viscosity. Micromachines.

[120] Antypas H., Zhang T., Choong F.X., Melican K., Richter-Dahlfors A. (2023). Dynamic single cell analysis in a proximal-tubule-on-chip reveals heterogeneous epithelial colonization strategies of uropathogenic *Escherichia coli* under shear stress. FEMS Microbes.

[121] Ejrnæs K. (2011). Bacterial characteristics of importance for recurrent urinary tract infections caused by Escherichia coli. Dan. Med. Bull..

[122] Ferrell N., Sandoval R.M., Molitoris B.A., Brakeman P., Roy S., Fissell W.H. (2019). Application of physiological shear stress to renal tubular epithelial cells. Methods Cell Biol..

[123] Nashimoto Y., Okada R., Hanada S., Arima Y., Nishiyama K., Miura T., Yokokawa R. (2019). Vascularized cancer on a chip: The effect of perfusion on growth and drug delivery of tumor spheroid. Biomaterials.

[124] Jiang L., Khawaja H., Tahsin S., Clarkson T.A., Miranti C.K., Zohar Y. (2024). Microfluidic-based human prostate-cancer-on-chip. Front. Bioeng. Biotechnol..

[125] Patra B., Peng C.-C., Liao W.-H., Lee C.-H., Tung Y.-C. (2016). Drug testing and flow cytometry analysis on a large number of uniform sized tumor spheroids using a microfluidic device. Sci. Rep..

[126] Zhang X., Karim M., Hasan M.M., Hooper J., Wahab R., Roy S., Al-Hilal T.A. (2022). Cancer-on-a-chip: Models for Studying Metastasis. Cancers.

[127] St-Georges-Robillard A., Masse M., Cahuzac M., Strupler M., Patra B., Orimoto A.M., Kendall-Dupont J., Péant B., Mes-Masson A.-M., Leblond F. (2018). Fluorescence hyperspectral imaging for live monitoring of multiple spheroids in microfluidic chips. Analyst.

[128] Rodriguez A.D., Horowitz L.F., Castro K., Kenerson H., Bhattacharjee N., Gandhe G., Raman A., Monnat R.J., Yeung R., Rostomily R.C. (2020). A microfluidic platform for functional testing of cancer drugs on intact tumor slices. Lab Chip.

[129] Flont M., Jastrzębska E., Brzózka Z. (2020). A multilayered cancer-on-a-chip model to analyze the effectiveness of new-generation photosensitizers. Analyst.

[130] Eduati F., Utharala R., Madhavan D., Neumann U.P., Longerich T., Cramer T., Saez-Rodriguez J., Merten C.A. (2018). A microfluidics platform for combinatorial drug screening on cancer biopsies. Nat. Commun..

[131] Aref A.R., Campisi M., Ivanova E., Portell A., Larios D., Piel B.P., Mathur N., Zhou C., Coakley R.V., Bartels A. (2018). 3D microfluidic *ex vivo* culture of organotypic tumor spheroids to model immune checkpoint blockade. Lab Chip.

[132] Jouybar M., de Winde C.M., Wolf K., Friedl P., Mebius R.E., Toonder J.M.D. (2023). Cancer-on-chip models for metastasis: Importance of the tumor microenvironment. Trends Biotechnol..

[133] Flont M.N., Dybko A., Jastrzębska E. (2023). A layered cancer-on-a-chip system for anticancer drug screening and disease modeling. Analyst.

[134] Vernetti L., Gough A., Baetz N., Blutt S., Broughman J.R., Brown J.A., Foulke-Abel J., Hasan N., In J., Kelly E. (2017). Functional Coupling of Human Microphysiology Systems: Intestine, Liver, Kidney Proximal Tubule, Blood-Brain Barrier and Skeletal Muscle. Sci. Rep..

[135] E Watson D., Hunziker R., Wikswo J.P. (2017). Fitting tissue chips and microphysiological systems into the grand scheme of medicine, biology, pharmacology, and toxicology. Exp. Biol. Med..

[136] Jang M., Kim H.N. (2023). From Single- to Multi-organ-on-a-Chip System for Studying Metabolic Diseases. BioChip J..

[137] Partners T.O., Mastrangeli M., Millet S., Raaij J.v.D.E.-V. (2019). Organ-on-chip in development: Towards a roadmap for organs-on-chip. Altex.

[138] Marzagalli M., Pelizzoni G., Fedi A., Vitale C., Fontana F., Bruno S., Poggi A., Dondero A., Aiello M., Castriconi R. (2022). A multi-organ-on-chip to recapitulate the infiltration and the cytotoxic activity of circulating NK cells in 3D matrix-based tumor model. Front. Bioeng. Biotechnol..

[139] Esch M., King T., Shuler M. (2011). The Role of Body-on-a-Chip Devices in Drug and Toxicity Studies. Annu. Rev. Biomed. Eng..

[140] Chen Y.-C., Lee K.-Y., Liao H.-J., Sun W.-L., Huang W.-C., Wang Y.-S., Chang W.-C., Liu C.-H. (2024). A tumor on a chip for studying immune-cell infiltration into tumor under chemo/immunotherapy treatments. Sens. Actuators B Chem..

[141] Li Z., Guo Y., Yu Y., Xu C., Xu H., Qin J. (2016). Assessment of metabolism-dependent drug efficacy and toxicity on a multilayer organs-on-a-chip. Integr. Biol..

[142] Wagner I., Materne E.-M., Brincker S., Süßbier U., Frädrich C., Busek M., Sonntag F., Sakharov D.A., Trushkin E.V., Tonevitsky A.G. (2013). A dynamic multi-organ-chip for long-term cultivation and substance testing proven by 3D human liver and skin tissue co-culture. Lab Chip.

[143] Maschmeyer I., Hasenberg T., Jaenicke A., Lindner M., Lorenz A.K., Zech J., Garbe L.-A., Sonntag F., Hayden P., Ayehunie S. (2015). Chip-based human liver–intestine and liver–skin co-cultures—A first step toward systemic repeated dose substance testing in vitro. Eur. J. Pharm. Biopharm..

[144] Shao J., Wu L., Wu J., Zheng Y., Zhao H., Jin Q., Zhao J. (2009). Integrated microfluidic chip for endothelial cells culture and analysis exposed to a pulsatile and oscillatory shear stress. Lab Chip.

[145] Zhang C., Zhao Z., Rahim N.A.A., van Noort D., Yu H. (2009). Towards a human-on-chip: Culturing multiple cell types on a chip with compartmentalized microenvironments. Lab Chip.

[146] Zhang W., Zhang Y.S., Bakht S.M., Aleman J., Shin S.R., Yue K., Sica M., Ribas J., Duchamp M., Ju J. (2016). Elastomeric free-form blood vessels for interconnecting organs on chip systems. Lab Chip.

[147] Maschmeyer I., Lorenz A.K., Schimek K., Hasenberg T., Ramme A.P., Hübner J., Lindner M., Drewell C., Bauer S., Thomas A. (2015). A four-organ-chip for interconnected long-term co-culture of human intestine, liver, skin and kidney equivalents. Lab Chip.

[148] Wang Y., Wang P., Qin J. (2021). Microfluidic Organs-on-a-Chip for Modeling Human Infectious Diseases. Acc. Chem. Res..

[149] Li Z.A., Tuan R.S. (2022). Towards establishing human body-on-a-chip systems. Stem Cell Res. Ther..

[150] Sánchez-Costa M., Urigoitia A., Comino N., Arnaiz B., Khatami N., Ruiz-Hernandez R., Diamanti E., Abarrategi A., López-Gallego F. (2024). In-Hydrogel Cell-Free Protein Expression System as Biocompatible and Implantable Biomaterial. ACS Appl. Mater. Interfaces.

[151] Pan S., Zhang Y., Natalia A., Lim C.Z.J., Ho N.R.Y., Chowbay B., Loh T.P., Tam J.K.C., Shao H. (2021). Extracellular vesicle drug occupancy enables real-time monitoring of targeted cancer therapy. Nat. Nanotechnol..

[152] Peng B., Tong Z., Tong W.Y., Pasic P.J., Oddo A., Dai Y., Luo M., Frescene J., Welch N.G., Easton C.D. (2020). In Situ Surface Modification of Microfluidic Blood–Brain-Barriers for Improved Screening of Small Molecules and Nanoparticles. ACS Appl. Mater. Interfaces.

[153] Yang L.W.Y., Ng W.Y., Lei X., Tan S.C.Y., Wang Z., Yan M., Pargi M.K., Zhang X., Lim J.S., Gunasekeran D.V. (2023). Development and testing of a multi-lingual Natural Language Processing-based deep learning system in 10 languages for COVID-19 pandemic crisis: A multi-center study. Front. Public Health.

[154] Srinivasu P.N., SivaSai J.G., Ijaz M.F., Bhoi A.K., Kim W., Kang J.J. (2021). Classification of skin disease using deep learning neural networks with MobileNet V2 and LSTM. Sensors.

[155] Boutin M.E., Hampton C., Quinn R., Ferrer M., Song M.J. (2019). 3D Engineering of Ocular Tissues for Disease Modeling and Drug Testing. Adv. Exp. Med. Biol..

[156] Shirure V.S., Hughes C.C., George S.C. (2021). Engineering Vascularized Organoid-on-a-Chip Models. Annu. Rev. Biomed. Eng..

[157] Liu H., Wang Y., Zhang X., Zhang M., Wang P., Shang J., Li Z., Gong L., Guo J., Sun W. (2024). Standard: Human intestine-on-a-chip. Cell Regen..

